# Outer Hair Cell and Auditory Nerve Function in Speech Recognition in Quiet and in Background Noise

**DOI:** 10.3389/fnins.2017.00157

**Published:** 2017-04-07

**Authors:** Richard Hoben, Gifty Easow, Sofia Pevzner, Mark A. Parker

**Affiliations:** ^1^Department of Otolaryngology, Steward St. Elizabeth's Medical CenterBoston, MA, USA; ^2^Department of Otolaryngology, Head and Neck Surgery, Tufts University School of MedicineBoston, MA, USA

**Keywords:** hidden hearing loss, QuickSIN, outer hair cell, auditory nerve, electrocochleography (ECochG), compound action potential (CAP), wave I auditory brainstem response (ABR), distortion product otoacoustic emission

## Abstract

The goal of this study was to describe the contribution of outer hair cells (OHCs) and the auditory nerve (AN) to speech understanding in quiet and in the presence of background noise. Fifty-three human subjects with hearing ranging from normal to moderate sensorineural hearing loss were assayed for both speech in quiet (Word Recognition) and speech in noise (QuickSIN test) performance. Their scores were correlated with OHC function as assessed via distortion product otoacoustic emissions, and AN function as measured by amplitude, latency, and threshold of the VIIIth cranial nerve Compound Action Potential (CAP) recorded during electrocochleography (ECochG). Speech and ECochG stimuli were presented at equivalent sensation levels in order to control for the degree of hearing sensitivity across patients. The results indicated that (1) OHC dysfunction was evident in the lower range of normal audiometric thresholds, which demonstrates that OHC damage can produce “Hidden Hearing Loss,” (2) AN dysfunction was evident beginning at mild levels of hearing loss, (3) when controlled for normal OHC function, persons exhibiting either high or low ECochG amplitudes exhibited no statistically significant differences in neither speech in quiet nor speech in noise performance, (4) speech in noise performance was correlated with OHC function, (5) hearing impaired subjects with OHC dysfunction exhibited better speech in quiet performance at or near threshold when stimuli were presented at equivalent sensation levels. These results show that OHC dysfunction contributes to hidden hearing loss, OHC function is required for optimum speech in noise performance, and those persons with sensorineural hearing loss exhibit better word discrimination in quiet at or near their audiometric thresholds than normal listeners.

## Introduction

It is clear that the audiogram, which is the standard metric of audition in humans, is inadequate in identifying otopathologies that contribute to hearing impairment (Moore, 2002; Makary et al., 2011; Liberman et al., 2016). In part, this is because of an incomplete understanding of the cellular basis of decoding complex stimuli, such as speech comprehension in the presence of background noise, and defining the functional roles of cochlear cell types involved in audition may lead to better clinical assessment. Speech recognition in the presence of background noise is a primary complaint of the hearing impaired, and auditory neuroscience seems to have come full circle regarding the understanding of the cellular basis of this function in the cochlea. As early as the 1950s, the auditory nerve (AN) was proposed to play the primary role in the ability to understand speech (Schuknecht and Woellner, 1953). This led to the development of the cochlear implant (House, 1974). However, the discovery of otoacoustic emissions in the 1970s (Kemp, 1978), and later discovery of the motile abilities of outer hair cells (OHCs) in the 1980s (Brownell et al., 1985), led to a paradigm shift in focus that OHCs play a primary role amplifying the speech signal for the fine tuning that is essential for understanding spoken language. OHC function has been described as both a cochlear amplifier (Davis, 1983), where OHCs amplify the passive motion of the basilar membrane (BM), and as a bank of frequency-specific filters that fine tune the acoustic signal (Goldstein et al., 1971; Ruggero, 1994). While these models are correct from a theoretical perspective, translating these functions to a clinical perspective is essential in our understanding of how OHC function contributes to audition. For example, whether OHCs function as cochlear amplifiers that amplify signals at threshold and/or a series of band-width filters to aid speech recognition in the presence of background noise is unknown.

More recently, evidence in animal models have re-examined the functional roles of the AN in quiet and in the presence of background noise (Kujawa and Liberman, 2006, 2009; Furman et al., 2013). Much of this work is based on the observation in animals that the AN is comprised of distinct populations of AN fibers based on their spontaneous firing rate (Liberman, 1978). AN fibers with low spontaneous rates function in increasing background noise, and AN fibers with high spontaneous rates function in quiet backgrounds at or near thresholds (Furman et al., 2013). Low-level noise exposure studies where normal OHC function has been preserved (Kujawa and Liberman, 2009; Lin et al., 2011) suggest that low spontaneous rate AN fibers are selectively damaged leaving high spontaneous rate fibers intact (Furman et al., 2013). The hypothesis derived from these studies is that if humans exhibit similar damage of the low spontaneous rate fibers, the ability to hear in complex listening situations such as speech in the presence of background noise would be diminished. Unfortunately, speech discrimination is very difficult to measure in laboratory animals, so confirmation of this hypothesis in humans has been a recent focus of investigation (Bramhall et al., 2015, 2017; Liberman et al., 2016; Prendergast et al., 2017).

Using loss of function data collected from normal and hearing impaired humans, the aim of this study was to describe the individual and combined contributions of OHCs and the AN in speech discrimination. OHC function was measured using distortion product otoacoustic emissions (DPOAEs), and AN function was measured using the amplitude, latency, and threshold of the VIIIth cranial nerve Compound Action Potential (CAP) measured during ECochG. These responses were correlated to human subject variables that included age, degree of sensorineural hearing loss (SNHL), as well as speech discrimination performances in quiet (SIQ) or in the presence of competing background noise (SIN). Previous research has demonstrated that SNHL has a strong correlation with both SIN performance and CAP amplitude (Bramhall et al., 2015). In order to control for the degree of SNHL, the stimuli for speech testing and AN analyses were presented in the sensation level (SL) scale, which incorporates an individual's threshold as the reference for the intensity scale of the stimuli.

The results demonstrate that OHC dysfunction is detected in the normal diagnostic range of a standard audiogram, optimum SIN performance is correlated with OHC function, and those persons with SNHL exhibit better word discrimination in quiet at or near their audiometric thresholds than normal listeners. The results are best described using linear systems theory where OHCs function as a bank of frequency and intensity filters. These results not only help define to cellular basis of audition, but will also focus the direction of future hearing loss therapies.

## Materials and methods

### Subjects

Fifty-three English speaking adults (14 males and 39 females) age range 22–71 (mean of 46.0 years old) were recruited from our clinic at St. Elizabeth's Medical Center in Boston, MA to participate in this study. All study procedures were performed and approved by the St. Elizabeth's Institutional Review Board and all participants in the study provided informed consent. An audiological evaluation including tympanometry, air and bone conduction thresholds, speech reception threshold (SRT), and Word Recognition in Quiet using NU-6 word lists was completed for each subject. The inclusion criteria consisted of a high-frequency pure tone average (hfPTA = mean of thresholds at 1, 2, and 4 kHz) of 50 dB HL or less, normal (Type A) tympanometry using a 226 Hz probe tone (Jerger et al., 1972), no conductive pathology, no pure tone asymmetry >10 dB HL between ears, and no documented otological disease. All of the following measurements were recorded from the best ear based on their hfPTA. The entire procedure took ~2 h and most subjects broke these into two 1 h sessions.

### Audiometry

A Madsen Astera audiometer was used to generate the pure tone and speech stimuli and the responses were recorded on GN Otometrics Otosuite V 4.70.00 software. Behavioral threshold was obtained at 0.025, 0.05, 1, 2, 3, 4, 6, and 8 kHz using a modified Hughson-Westlake procedure (Carhart and Jerger, 1959) in 5 dB HL steps under a calibrated insert earphone in a audiometric sound booth. SRT using recorded materials was obtained this same procedure using spondee words rather than pure tone stimuli.

### Word recognition score (WRS) in quiet

Subjects were presented with a unique and randomized NU-6 wordlist (25 words) using recorded materials presented at 0, 10, 20, and 40 dB Sensation Level (SL; above SRT) under headphones in quiet in an audiometric sound booth and the percent of correct responses were recorded for each presentation level.

### Quick speech-in-noise (QSIN)

Quick Speech-In-Noise (QSIN) test (Killion et al., 2004) was used to asses speech recognition in the presence of background noise. Sentences were presented at 0, 10, 20, and 40 dB SL (relative to SRT) in the presence of multi-talker babble varying in signal-to-noise (SNR) ratio from 0 to 25 dB. HL Subjects were familiarized with the task using one practice list and then presented with 2 scored lists for each ear. Scores were averaged and reported as mean SNR loss, with larger positive numbers indicating poorer performance.

### Distortion product otoacoustic emission (DPOAE)

Distortion Product Otoacoustic Emission (DPOAE) amplitudes and thresholds were evoked using a Madsen Capella II Otoacoustic system and recorded using Otosuite software (version 4.70.00). DPOAE SNRs were measured using an 8 to 1 kHz F2 frequency sweep where L1 was set to 65 dB SPL and L2 was set to 55 dB SPL (F2/F1 ratio = 1.22; Kujawa and Liberman, 2009). The acceptance criterion was set to minimum DPOAE level of −5 dB SPL and SNR of 6 dB SPL or more. These recordings were repeated three times, and DPOAE SNRs were averaged to obtain mean SNR amplitude per F2. DPOAE thresholds were obtained using a 75 dB SPL to 25 dB SPL (L1 = L2) intensity sweep in 5 dB SPL steps at audiometric frequencies using the same acceptance criteria. Threshold was defined as the lowest intensity that elicited a DPOAE above the noise floor. Threshold was set at 20 dB SPL in cases where the DPOAE was present at the lowest presentation level (25 dB SPL), and was set to 80 dB SPL in cases where there was no repeatable DP present at the highest presentation level (75 dB SPL).

### Electrocochleagraphy (ECochG)

**Figure 1:**
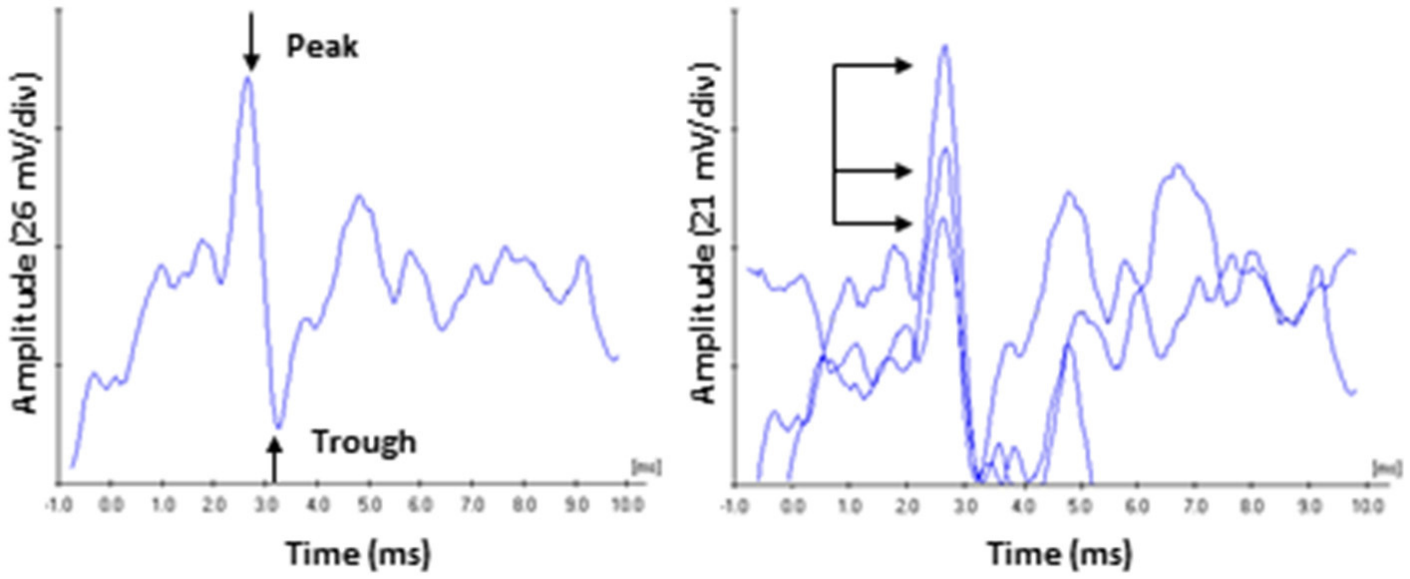
**ECochG recording of the compound action potential. (Left)** Representative tracing of a 60 dB SL presentation to a subject in the Normal Hearing group. Amplitude was measured from the peak of CAP to the following trough. **(Right)** Responses were recorded three times, and the average peak amplitudes and latencies were calculated. Arrows highlight the variability on amplitude characteristic of this recording in unanaesthetized human subjects. By comparison, CAP peak latency values (read off the abscissa) exhibited less variability.

Electrocochleagraphy (ECochG) waveforms were obtained using the Bio-logic Navigator Pro auditory evoked potentials system and incorporating Lilly wick tympanic membrane electrodes (Intelligent Hearing Systems) coupled with Bio-logic insert earphones. Electrodes were soaked in sterile normal saline solution at room temperature for 20 min, and then inserted into the external auditory meatus by an experienced audiologist so that the electrode rested on the tympanic membrane. An insert earphone was then placed in the same ear canal to deliver the acoustic stimuli and help stabilize the electrode. The reference electrode was place on the contralateral mastoid and the ground electrode was place on the high forehead (horizontal montage). An alternating polarity 4,000 Hz toneburst stimulus (Blackman ramp with a four cycle rise and fall) was presented at a repetition rate of 13.3/s with a 10–1,500 Hz filter and an amplifier gain of 50,000 and digitized in a 10.66 ms time window. The average waveform was generated from 1,000 sweeps. Acoustic stimuli were presented at 0, 30, 40, 50, 60, and 70 dB SL (in dB nHL relative to the hfPTA). Since behavioral detection thresholds are 25 dB lower than ABR thresholds (Ngan and May, 2001; Henry et al., 2011), the choice was made to base the SL scale on audiometric thresholds rather than ABR thresholds. Average ECochG waveforms were analyzed by an experienced audiologist with a clinical Certification in Neurophysiological Interoperate Monitoring (CNIM). The CAP was identified as the largest peak occurring at ~2.0–3.5 ms after stimulus onset and the amplitude was measured with the Bio-logic Auditory Evoked Potential software (version 6.2.0) as the difference in voltage between the peak of the CAP and the following trough (Lasky, 1984; Bramhall et al., 2015). At least three waveforms were generated for each ear and the average amplitudes, latencies, and thresholds for each presentation level were obtained and used for further analysis (Figure 1). The lowest presentation level to elicit a repeatable CAP was defined as threshold.

### Linear mixed effects modeling

The collected data was modeled as described in detail by Bramhall et al. (2015), with the exception that this current paper used SPSS (IBM SPSS Statistics version 23, release 23.0.0.0) rather than R to generate the models. Deidentified subject number was used as the random effects variable; covariates included subject age, DPOAE amplitudes and thresholds at all F2 frequencies, CAP amplitudes and latencies at all presentation levels, and CAP thresholds; the subjects hfPTA was used as the residual weighted variable; and the subjects QSIN scores were used as the dependent variable.

### Analysis

After data collection, patient responses were rank ordered and divided into groups as described in the text. Power analyses using an alpha of 0.05 determined the power to be >0.8 for analyses between the groups described in the text. A test of normalcy indicated that these results were not normally distributed so non-parametric statistical analyses were utilized. Correlations between group variables were conducted using Kendall's tau-b (τ_b_) correlation coefficient, which is a non-parametric measure of the strength and direction of an association between variables ranked in either ordinal or continuous scales using SPSS. The τ_b_ correlation coefficient was calculated for each condition (i.e., presentation level, frequency) as described, however only the strongest correlations were described in the text for clarity. SPSS also calculates the *p*-value of the τ_*b*_ correlation coefficient, which are plotted in appropriate figures. With the exception of the word recognition in quiet analyses, statistically significant trends between groups were measured by the non-parametric Jonckheere–Terpstra (J–T) test (Bewick et al., 2004). For clinically significant differences of word recognition in quiet, statistically significant differences in performance were based on previously published binomial modeling of word recognition scores (Thornton and Raffin, 1978). Three graphical methods are used to visualize the data in the main text. Data are plotted either as scatter plots of individual data points for correlational analysis; box and whisker plots using upper and lower quartiles (upper and lower ends of the box), median (line within the box), range of scores (error bars), and suspected outliers (either less than the lower quartile or higher than the upper quartile by 1.5 times the inter quartile range, open circles accompanied by subject identification number) in order to better visualize the variance within each group; or mean values with error bars representing the standard error of the mean to visuals the statistically significant differences between groups. For all figures, asterisks represent a *p* < 0.05.

## Results

### SNHL is correlated with SIN

**Figure 2:**
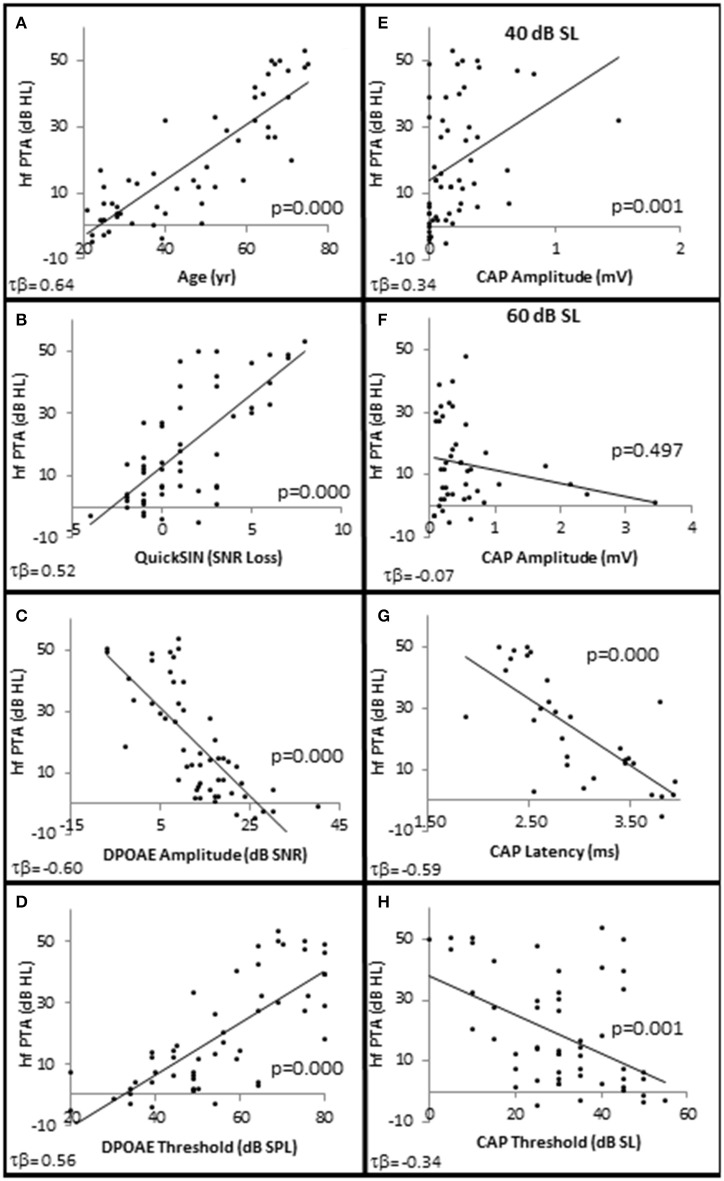
**SNHL is Correlated with age, speech in noise performance, and OHC function**. Distribution of SNHL (hfPTA) as a function of age **(A)**, SIN performance (Quick SIN) with better performance in noise corresponding to lower values of SNR loss **(B)**, OHC function measured by DPAOE amplitude **(C)**, and DPAOE threshold **(D)** at 4 kHz, AN function measured by CAP amplitude in response to 4 kHz tone pips presented at 40 dB SL **(E)** and 60 dB SL **(F)**, CAP latency in response to 4 kHz tone pips presented at 30 dB SL **(G)** and CAP thresholds **(H)**. Lines represent best fit (linear).

**Figure 3:**
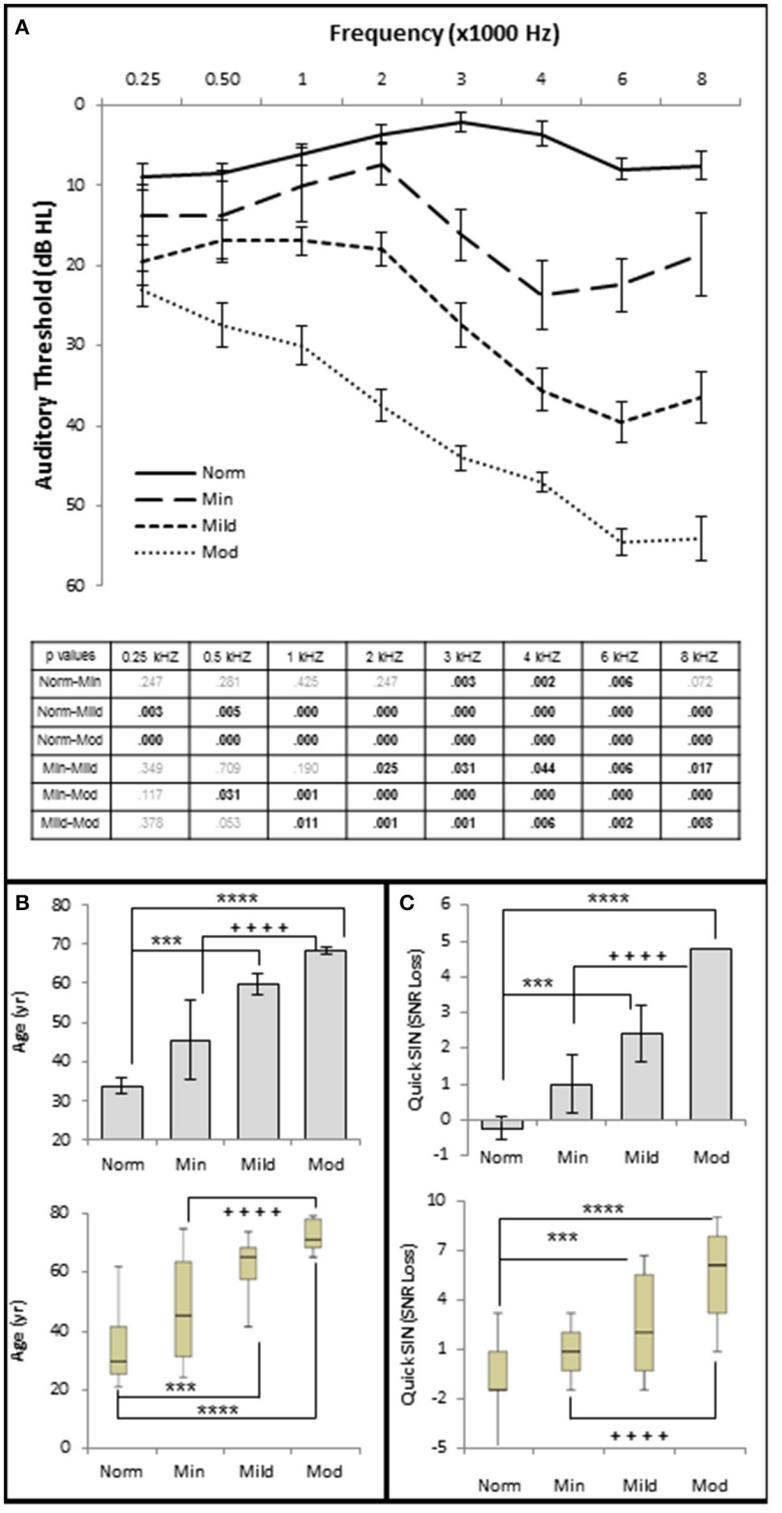
**Increasing SNHL is correlated with decreased speech in noise performance. (A)** Mean pure tone audiograms from each group. The table lists the *p*-values between each group by stimulus frequency. Bold text indicates a *p* < 0.05. **(B)** Distribution of the subject age (years) in each group. Top graph plots mean values +/1 s.e.m. Bottom graph plots this same data using upper and lower quartiles (box), median values (line within box), maximum and minimum scores (error bars). **(C)** Speech in noise performance from each group where lower SNR Loss corresponds to better performance in the presence of background noise. Top graph plots mean values ± 1 s.e.m. Bottom graph plots this same data using box and whisker plots. Norm, Normal Hearing Group; Min, Minimal SNHL; Mild, Mild SNHL; Mod, Moderate SNHL; ^***^ statistically significant difference between Normal and Mild SNHL groups; ^****^statistically significant difference between Normal and Moderate SNHL groups; ^++++^statistically significant difference between Minimal SNHL and Moderate SNHL groups.

The results show statistically significant correlations between SNHL (measured by hfPTA) and subject age, SIN performance (measured by QSIN SNR Loss), and OHC function (measured by DPAOE amplitude and threshold; Figure 2). In order to visualize these correlations, subjects were ranked by hfPTA and divided into one of four groups based on their degree of high frequency SNHL (Normal Hearing <15 dB HL, *n* = 7 males and 22 females; Minimal SNHL = 15–24 dB HL, *n* = 1 male and 3 females; Mild SNHL = 25–39 dB HL, *n* = 6 males and 4 females; Moderate SNHL = 40–50 dB HL, *n* = 6 males and 4 females; Figure 3). In clinical audiometry, the Minimal SNHL group represents the lower end of the Normal range and is most often used in pediatric rather than adult audiometry. There was a statistically significant positive correlation between SNHL (hfPTA) and age (τb = 0.636, *p* = 0.000; Figure 2A). The non-parametric J–T test for ordered alternatives showed that there was a statistically significant trend of increased age with increasing hearing loss (Figure 3B). Specifically, there was a statistically significant increase in age between the Normal hearing (33.7 ± 1.97 years) and Mild (59.6 ± 2.79 years) and Moderate SNHL groups (*p* = 0.00).

**Figure 4:**
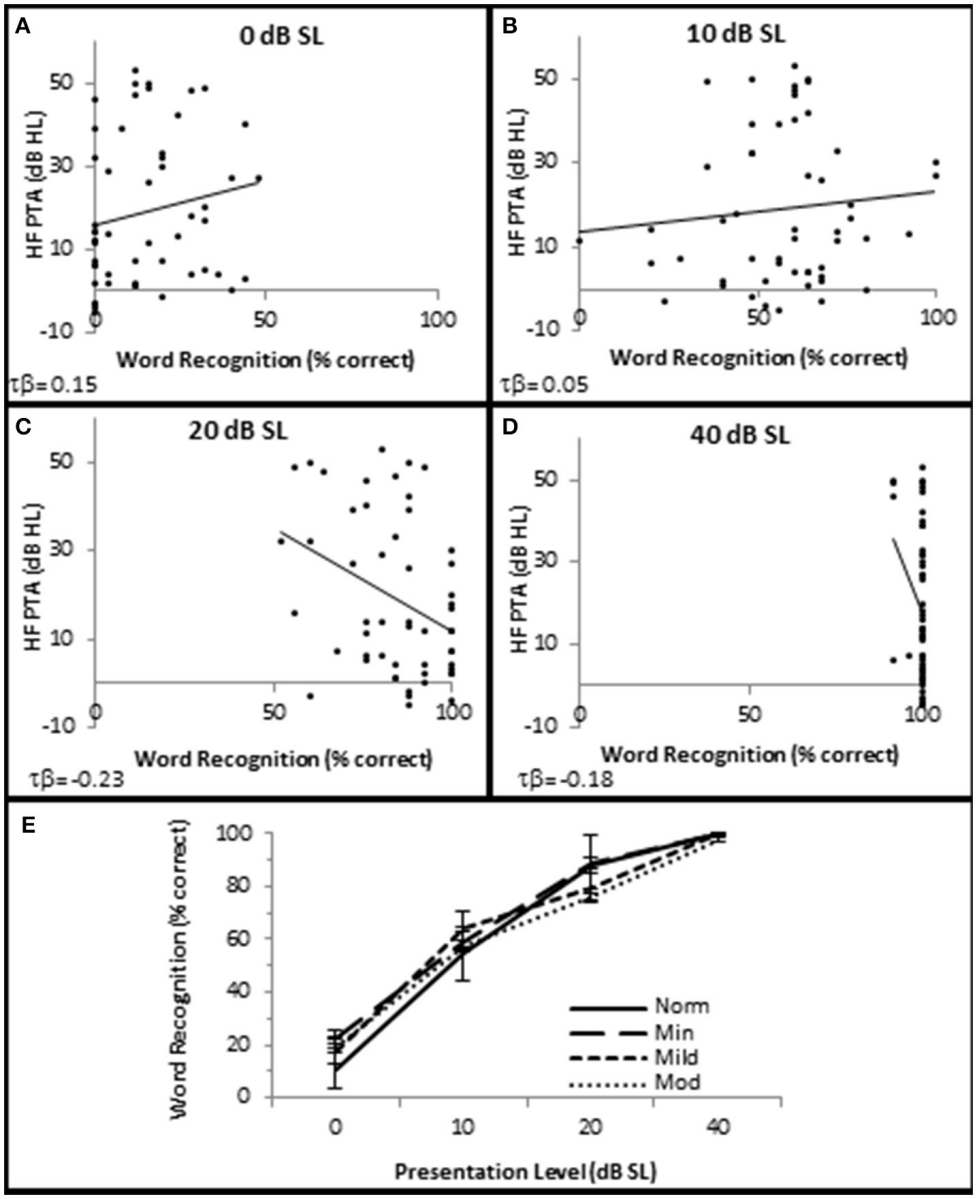
**SNHL is not clinically correlated with speech in quiet performance**. Distribution of individual WRS scores plotted as a function of SNHL (hfPTA) for 0 **(A)**, 10 **(B)**, 20 **(C)**, and 40 dB **(D)** sensation levels (dB above SRT). **(E)** Performance-intensity functions plotting the mean data from each SNHL group. Error bars = s.e.m. Norm, Normal Hearing Group; Min, Minimal SNHL; Mild, Mild SNHL; Mod, Moderate SNHL. Lines represent best fit (linear). Clinically statistically significant differences were based on Thornton and Raffin (1978).

SIQ testing between 10 and 40 dB SL showed no clinically significant differences between any of these groups (Figure 4). Interestingly, persons with normal hfPTAs exhibited a decrease on WRS (11.0 ± 2.57% correct) compared to subjects with moderate SNHL (19.6 ± 2.57% correct) when the word lists were presented at or near threshold (0 dB SL). While this difference was not significant on a clinical level, this trend will be explored in detail below.

There were no statistically significant differences in SIN testing between any groups analyzed in this study for 0, 10, and 20 dB SL presentation levels, so only 40 dB SL presentation levels are shown in the following figures. Similar to SIQ, SIN testing at 40 dB SL also showed a statistically significant direct correlation between hfPTA and QSIN score (τ_b_ = 0.518, *p* = 0.000; Figure 2B). J–T testing showed subjects in the Mild (2.4 ± 0.79 SNR loss, *p* = 0.002) and Moderate (and 4.8 ± 0.49 SNR loss, *p* = 0.000) SNHL groups exhibited statistically significant higher QSIN scores than persons in the Normal hfPTA group (−0.2 ± 1.08 SNR loss; Figure 3C). There was also a statistically significant increase in QSIN scores between the Minimal (1.0 ± 0.82 SNR loss) and Moderate SNHL groups (*p* = 0.007). Since higher QSIN scores represent poorer SIN performance (Killion et al., 2004), these results suggest that SIN performance worsens as hfPTA increases.

### Characteristics of OHC dysfunction in SNHL

**Figure 5:**
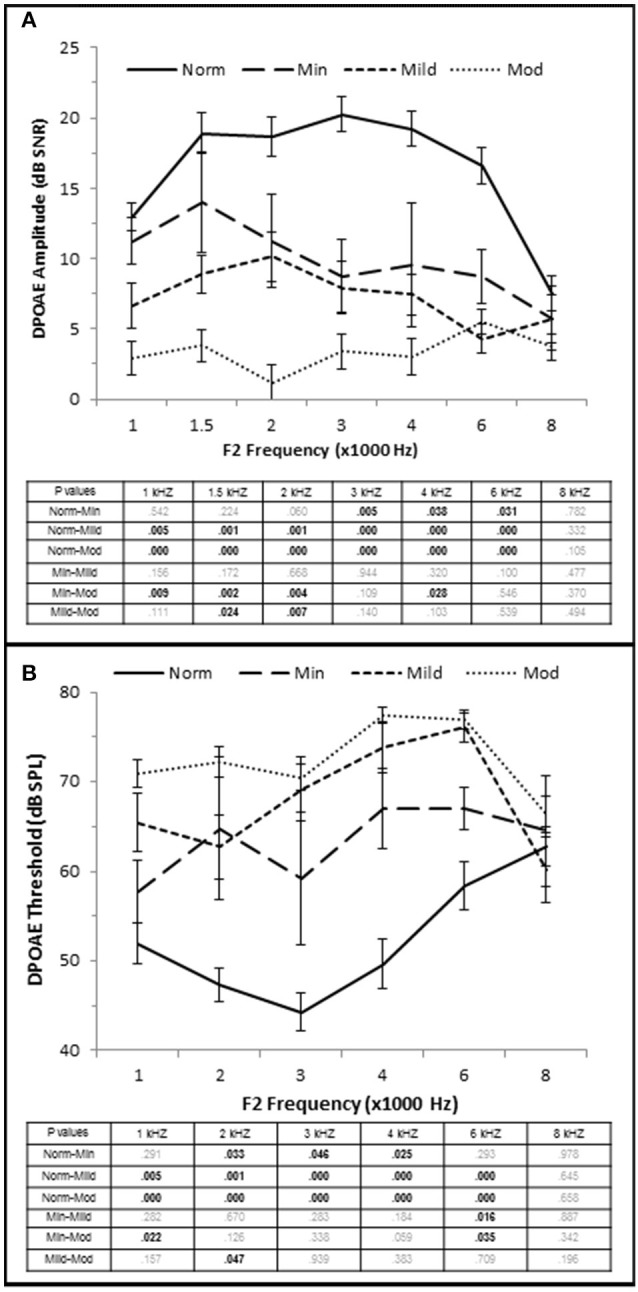
**OHC dysfunction occurs in minimal to moderate SNHL. (A)** DPOAE SNR (amplitude) and threshold **(B)** from each group. The tables list the *p*-values between each group by stimulus frequency. Bold text indicates a *p* < 0.05. Norm, Normal Hearing Group; Min, Minimal SNHL; Mild, Mild SNHL; Mod, Moderate SNHL.

OHC function was also correlated with the degree of SNHL. High frequency PTA was negatively (inversely) correlated with DPOAE amplitude (measured as DPAOE SNR) with a maximum correlation value at 4 kHz (τ_b_ = −0.601, *p* = 0.000; Figure 2C). J–T testing showed that even subjects in the Minimal SNHL group exhibited statistically significant decreases in DPOAE SNR at 3–6 kHz compared to subjects in the Normal hfPTA group (Figure 5A). Furthermore, there was a statistically significant decrease in DPOAE SNR as the degree of SNHL progressed from the Normal group at 1–6 kHz. The largest decrease in amplitude between consecutive groups occurred between the Normal hfPTA and Minimal SNHL groups (−11.49 dB SNR at 4 kHz). Similarly, subjects in the Moderate SNHL group exhibited statistically significantly diminished DPOAE SNRs compared to subjects in the Minimal group at 1–2 and 4 kHz, and to the Mild SNHL group at 1.5–2 kHz (*p*-values are listed in Figure 5A).

High Frequency PTA was directly correlated with DPOAE threshold with the strongest correlation coefficient at 3 kHz (τ_b_ = 0.564, *p* = 0.000; Figure 2D), and J–T testing showed an elevation on DPOAE threshold as hfPTA increased (Figure 5B). The Minimal SNHL group exhibited a statistically significant DPOAE threshold shift at 2–4 kHz compared to the Normal hfPTA group. Although the DPOAE threshold elevation progressed through the Mild and Moderate groups, the largest statistically significant threshold shift between consecutive groups occurred between the Normal and Minimal groups (17.38 dB SPL at 4 kHz).

Taken together, these results suggest that OHC function is correlated with pure tone audiometry, and that even subjects with minimal high frequency SNHL may exhibit statistically significant OHC damage. Since a PTA between 15 and 25 dB HL is often considered within the normal range in adult humans, this finding illustrates an example of an otopathology undetected in a standard audiogram commonly known as “Hidden Hearing Loss.”

### Characteristics of AN dysfunction in SNHL

**Figure 6:**
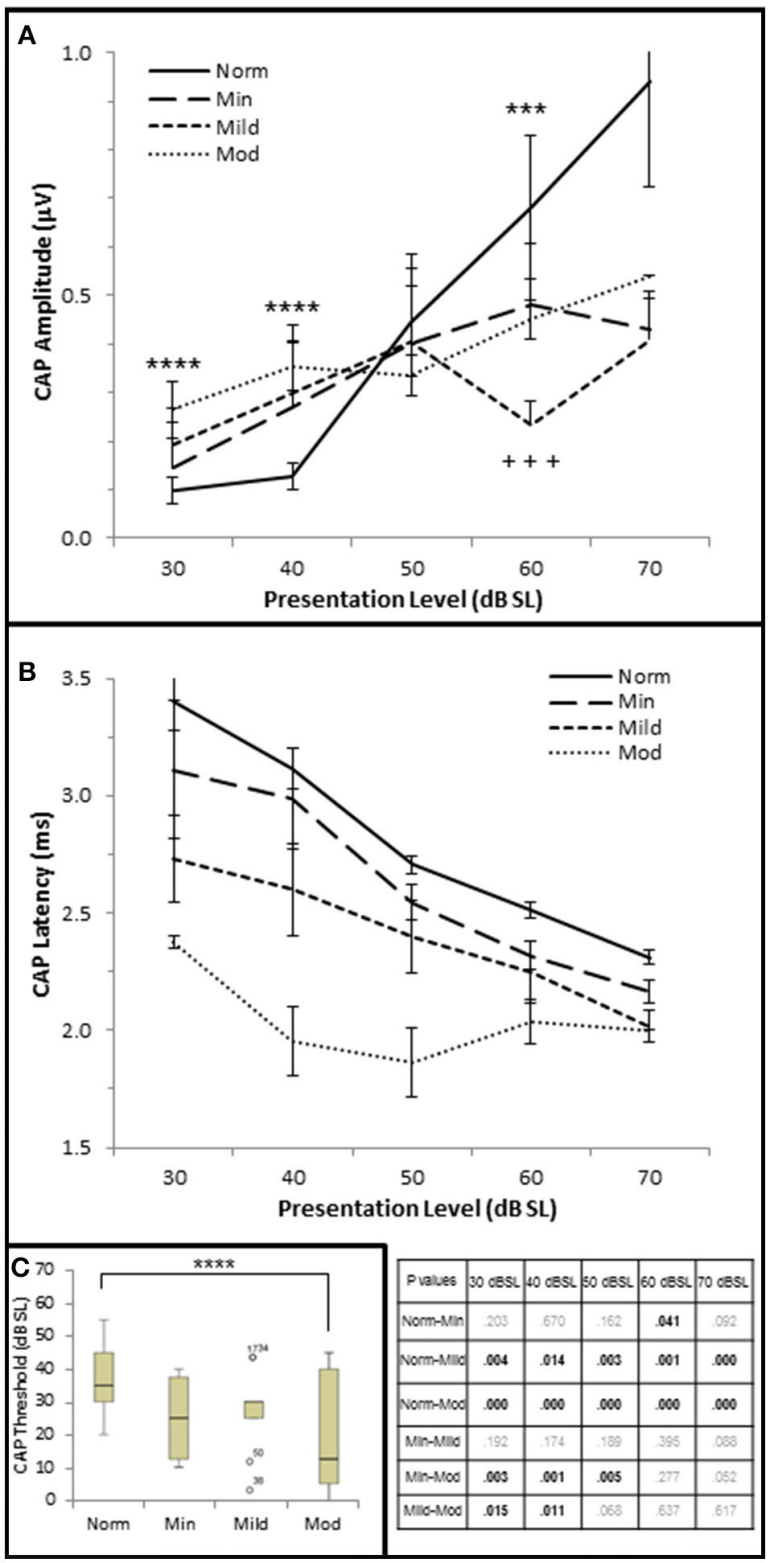
**AN dysfunction occurs in mild to moderate SNHL. (A)** Mean CAP amplitude from each group based on presentation level (dB SL). **(B)** CAP latency-intensity functions from each group. The table lists the *p*-values of CAP latency between each group by stimulus frequency. Bold text indicates a *p* < 0.05. **(C)** Box and whisker plots illustrating the median and range values of CAP thresholds for each group. Open circles represent suspected outliers, and numbers indicate the subject identification of the suspected outlier. Norm, Normal Hearing Group; Min, Minimal SNHL; Mild, Mild SNHL; Mod, Moderate SNHL; ^***^statistically significant difference between Normal and Mild SNHL groups; ^****^statistically significant difference between Normal and Moderate SNHL groups; ^+++^statistically significant difference between Minimal SNHL and Mild SNHL groups.

Next, CAP amplitude, latency, and thresholds were analyzed to study AN function within these groups. There was a direct correlation between hfPTA and CAP amplitude when 4 kHz tone pips were presented at 30 dB SL (τ_b_ = 0.209, *p* = 0.038; data not shown) and 40 dB SL (τ_*b*_ = 0.336, *p* = 0.001; Figure 2E) presentation levels. However, there was a difference in amplitude-intensity function between these groups (Figure 6A). The Normal hfPTA group exhibited small CAP amplitudes at low stimulus presentation levels (30–40 dB SL; *R*^2^ = 0.82, y = 0.0708x, intercept = 0.00) and a steep growth function in amplitude at 50–70 dB SL (*R*^2^ = 0.87, y = 0.3307x, intercept = 0.00) at higher presentation levels. In contrast, subjects in the Minimum, Mild, and Moderate SNHL groups exhibited a steeper growth function at low intensity levels, and flatter growth function at 50 dB SL and above. J–T testing between groups showed that presentation levels below 50 dB SL elicited progressive increases in CAP amplitude between Normal hfPTA and Moderate SNHL groups. Specifically, the Moderate SNHL group exhibited a statistically significant (*p* = 0.033) 0.17 μV increase over the Normal hfPTA group at 30 dB SL, and a statistically significant (*p* = 0.003) 0.23 μV increase over the Normal hfPTA group at 40 dB SL. Interestingly, tone pip presentation levels >50 dB SL resulted in a statistically non-significant (τβ = −0.072, *p* = 0.497; Figure 2F) trend in the opposite direction whereby the hearing impaired groups exhibited diminished CAP amplitudes compared to those in the Normal hfPTA group (Figure 6A). As can be seen by the error bars in Figure 6A, the group with better hearing exhibited increased amplitude variability at louder presentation levels. The only statistically significant difference between groups at louder presentation levels occurred at 60 dB SL between the Normal hfPTA and Mild SNHL groups (−0.45 μV difference, *p* = 0.003) and between Minimum and Mild SNHL groups (−0.23 μV difference, *p* = 0.045). Also unlike lower presentation levels, there was not a graded decrease in amplitude as a function of hfPTA at presentation levels above 50 dB SL. This may be due to the fact that many persons in the Moderate SNHL group had hfPTAs so great that the stimuli could either not be generated at such a high level (i.e., 105 dB SPL), or that these presentation levels were intolerably loud for the subjects.

In contrast to the variability observed in CAP amplitude, CAP latency-intensity functions exhibited a more consistent trend across groups. High frequency SNHL correlated with a statistically significant decrease in CAP latency at all presentation levels, with a maximum correlation coefficient at 30 dB SL (τ_b_ = 0–0.592, *p* = 0.000; Figure 2G). The J–T test (Figure 6B) showed that CAP latency-intensity functions exhibited a progressive and statistically significant decrease between Normal hfPTA groups and Mild SNHL (maximum latency shift at 30 dB SL of 0.67 ms, *p* = 0.004), and between Normal hfPTA and Moderate SNHL groups (maximum latency shift at 40 dB SL of 1.17 ms *p* = 0.000) at all intensity levels. Similarly, there was a statistically significant decrease in latency-intensity functions at lower levels of intensity between the Minimal and Moderate SNHL groups (−0.74 ms at 30 dB SL, *p* = 0.003; −1.04 ms at 40 dB SL, *p* = 0.001; −0.69 ms at 50 dB SL, *p* = 0.001), and the Mild and Moderate SNHL groups (−0.36 ms at 30 dB SL, *p* = 0.015; −0.65 ms at 40 dB SL, *p* = 0.011).

In addition to CAP amplitude and latency, there was a statistically significant inverse correlation between hfPTA and CAP threshold (τ_b_ = −0.343, *p* = 0.001; Figure 2H). J–T testing showed a statistically significant decrease in CAP threshold between the Normal hfPTA and Moderate SNHL groups (−15.7 dB SL difference, *p* = 0.011; Figure 6C). Although there was a significant correlation of decreased CAP threshold with high frequency SNHL, no other groups exhibited statistically significant differences between them.

Contrary to stimuli played at the same overall level dB SPL, stimuli played at equivalent SPL respective to pure tone average show that increased hearing loss leads to a general trend of lower CAP thresholds, shorter CAP latencies, and smaller CAP amplitudes at low presentation levels. At higher presentation levels, there was a general trend that hearing loss resulted in the expected results of decreases CAP amplitudes, however the trend that SNHL correlated with shorter CAP latencies was still evident. It should be noted that unlike the DPOAE results, there were no significant differences between the Normal hfPTA and Minimal SNHL groups in terms of CAP amplitude or threshold. In terms of CAP latency, the only statistically significant difference between these two groups was a −0.19 μV (*p* = 0.041) decrease that occurred at 60 dB SL presentation levels. Given these results, it is difficult to say there is a statistically significant difference between Normal hfPTA and Minimal SNHL in terms of AN activity. However, there is a statistically significant graded decrease in latency as SNHL increases from Mild to Moderate severity.

### SIN is positively correlated with OHC function

**Figure 7:**
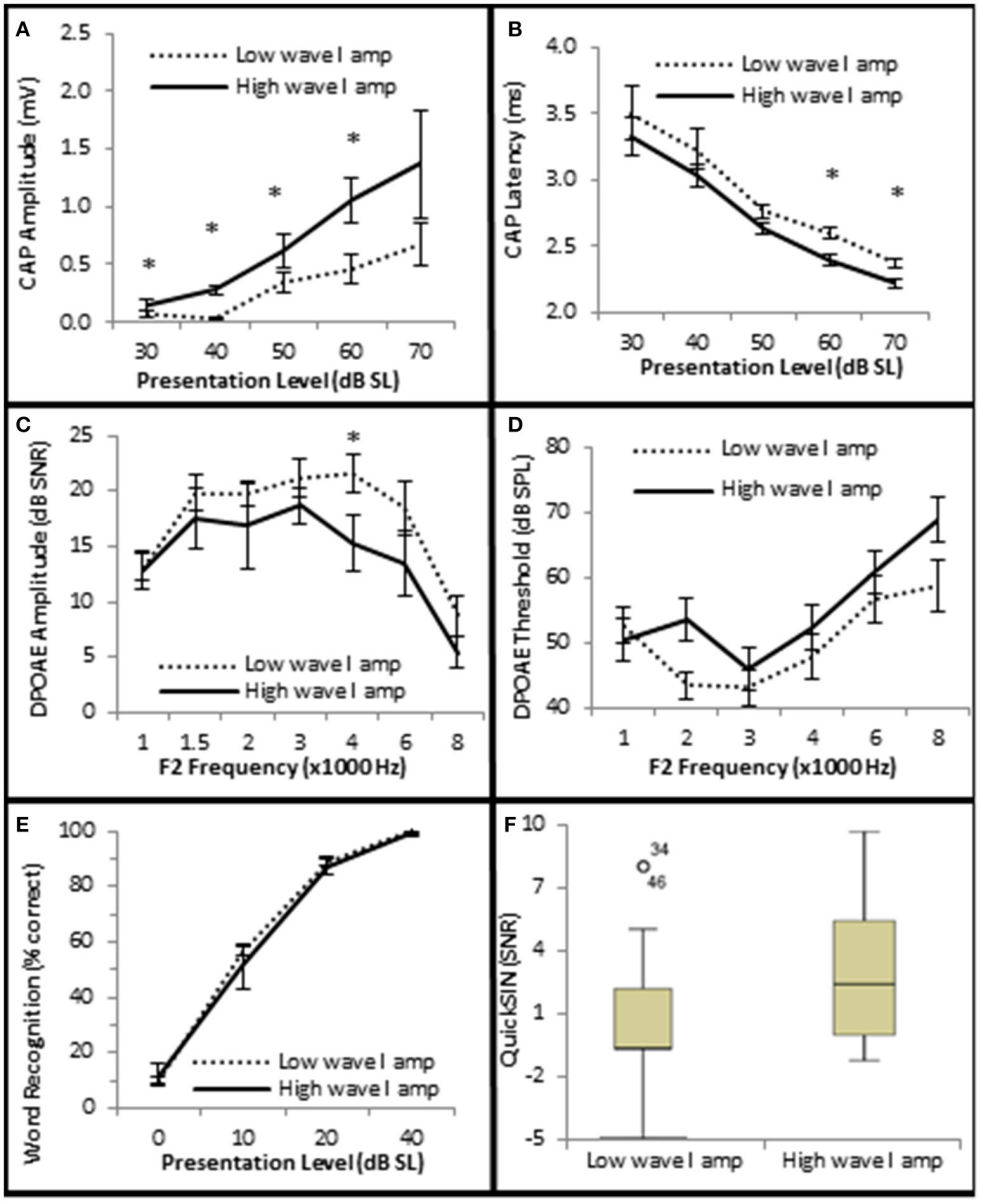
**Subjects with larger CAP amplitudes exhibited no significant improvement in SIQ or SIN when OHC function is normal. (A)** Persons exhibiting normal OHC function (Normal Group) were subdivided into two groups (Low and High) based on whether their CAP amplitudes were either higher or lower than the group mean ± 1 s.e.m. Persons exhibiting normal OHC function and high CAP amplitudes exhibited statistically significant shorter CAP latencies at high presentation levels **(B)**, but failed to exhibit statistically significant differences in DPOAE SNRs **(C)** or DPOAE thresholds **(D)** at most frequencies. Speech testing showed no clinically significant differences in word recognition in quiet **(E)** or speech recognition ion the presence of background noise **(F)** between these groups. Panels **(A–E)** represent mean data +/1 1 s.e.m. Panel **(F)** is a box and whisker plot showing the median data (line within the box). Open circle represents suspected outliers, and numbers indicate the subject identification of the suspected outlier. ^*^Statistically significant difference between groups.

Next, the data was analyzed to determine whether AN density, measured by CAP amplitude (Kujawa and Liberman, 2009), contributed to SIN performance. Since diminished DPOAE SNRs and increased DPOAE thresholds existed in the Minimal-Moderate SNHL groups (Figure 5B) only the Normal group (*n* = 29) was used in this analysis in order to control for OHC loss. The Normal group was ranked by CAP amplitudes at the 40 dB SL presentation level, and divided into high and low CAP amplitude groups based on whether their CAP amplitudes were either 1 s.e.m higher or 1 s.e.m lower than the Normal SNHL group mean of 156 μV (Low CAP < 156 μV < High CAP; Figure 7A). J–T testing indicated that persons with normal hfPTAs and normal OHC function who also exhibited higher CAP amplitudes exhibited statistically significantly shorter CAP latencies at higher presentation levels (maximum difference of −0.206 ms, *p* = 0.002; Figure 7B). This data suggest a general trend of an inverse relationship between CAP amplitude and latency at both low and high presentation levels when OHC function is normal. The data further showed there was no statistically significant differences in DPOAE SNRs between these two groups at most frequencies, however there was a statistically insignificant difference in DPOAE SNR at 4 k Hz (−5.1 dB SPL difference at 40 dB SL, *p* = 0.048; Figure 7C), while there were no statistically significant differences in DPOAE threshold between these groups (Figure 7D). Next, SIN and SIQ scores between these groups were analyzed to determine whether AN function played a solitary role in speech recognition. There were no significant differences in either SIQ (Figure 7E) or SIN (Figure 7F) performance in persons with diminished CAP amplitudes and normal OHC function. This data suggests that AN function by itself does not play a significant role in speech recognition in quiet or in the presence of background noise.

**Figure 8:**
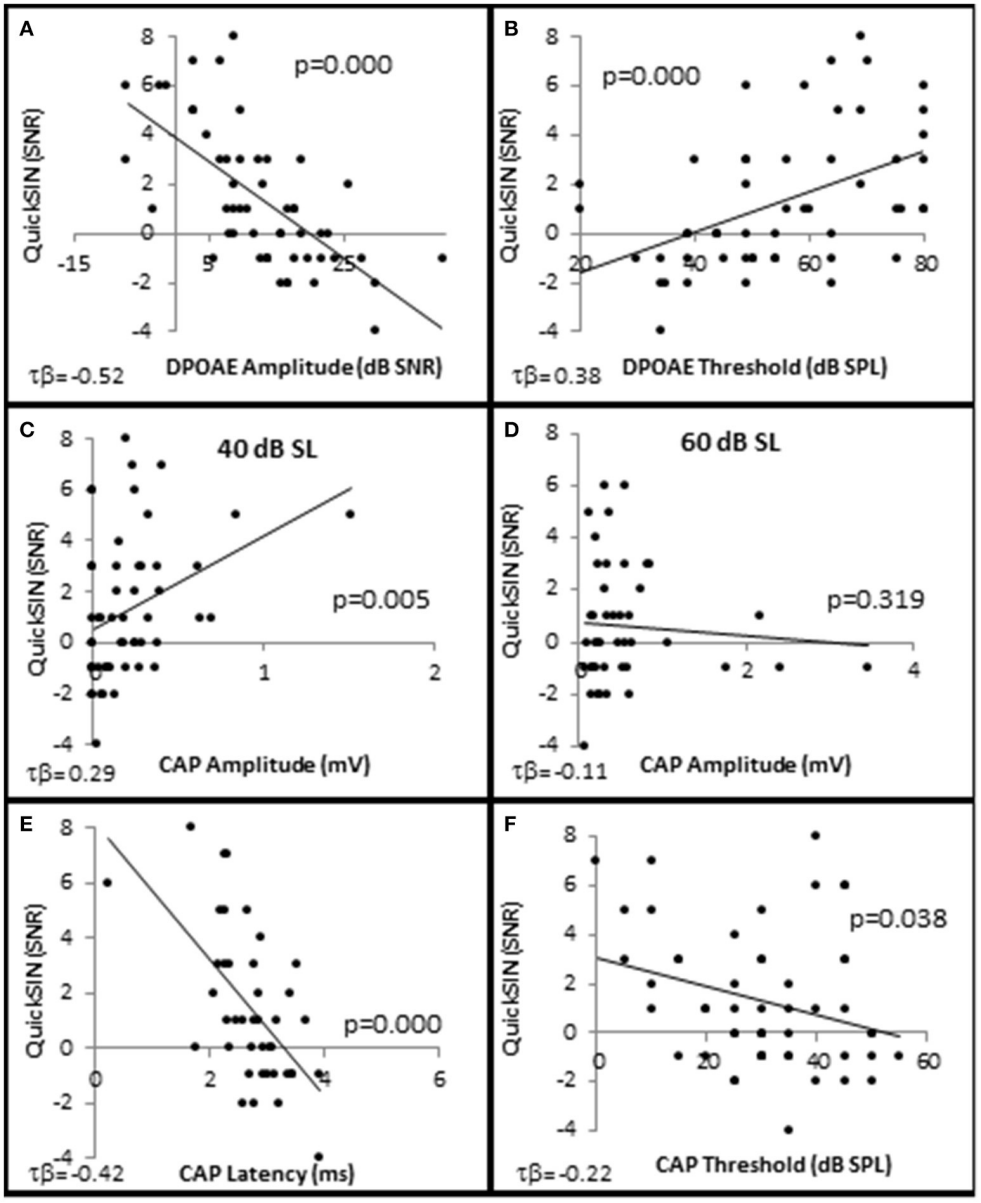
**Speech in noise performance is correlated with OHC function**. Distribution of individual QuickSIN scores plotted as a function DPAOE amplitude **(A)**, DPAOE threshold **(B)**, CAP amplitude when the 4 kHz tone pip is presented at 40 dB SL **(C)**, and 60 dB SL **(D)**, CAP latency at 40 dB SL **(E)**, and CAP threshold **(F)**. As noted in the text, all correlations were statistically significant with the exception of **(D)**. Lines represent best fit (linear).

In order to determine which cell types play a role in speech discrimination in the presence of background noise, all of the subjects from each group were used to correlate SIN performance with OHC and AN function (Figure 8). The results indicated that SIN performance was correlated with DPOAE function, where lower QSIN scores (better performance in noise) inversely correlated with DPOAE SNR (maximum τ_b_ = −0.522, *p* = 0.000 at 4 kHz; Figure 8A) and a directly correlated with DPOAE thresholds (maximum τ_b_ = 0.378, *p* = 0.000 at 3 kHz; Figure 8B). To further investigate these correlations, subjects were ranked by QuickSIN scores, and were divided into either Normal SIN (QSIN <1 dB SNR loss) or Poorer SIN (QSIN > 0 dB SNR loss) groups. It should be noted that the manufactures' QSIN cutoff score between normal and mild SIN impairment is 2 dB SNR loss, with 3 dB SNR loss representing “near normal.” However, the new data presented in Figure 5 demonstrates that OHC damage can occur in a person with a hfPTA as low as 15 dB HL, and Figure 3C shows that the QSIN cutoff for normal OHC function is −0.2 ± 0.3 dB SNR loss. Therefore, in order control for hidden hearing loss that was not accounted for by the manufactures of the QSIN, this paper will use a QSIN score of <1 dB SNR loss to differentiate between SIN performance in a non-pathological ear and a QSIN score > 0 dB SNR loss to correspond to an SIN performance in a pathological ear.

**Figure 9:**
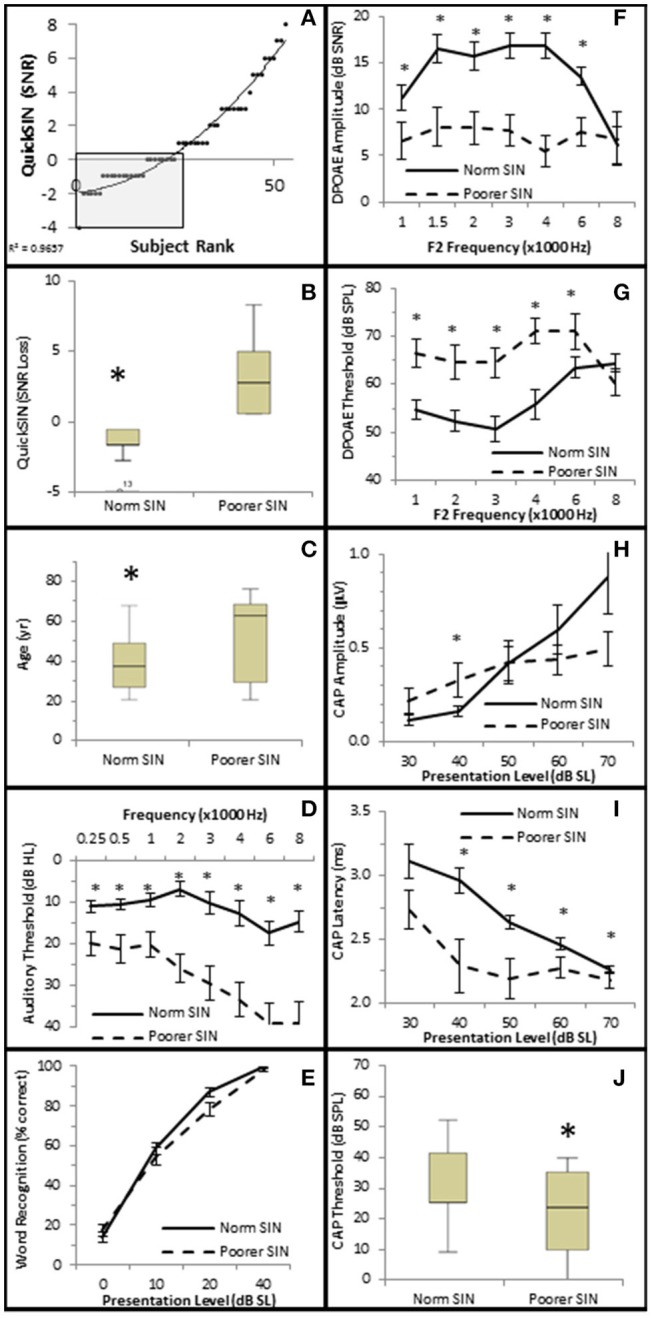
**Subjects performing better in noise were younger with better audiometric thresholds and better OHC functions. (A)** Subjects were ranked by QuickSIN scores, and divided into either Normal SIN (QSIN <1 dB SNR loss, shaded box) or Poorer SIN (QSIN > 0 dB SNR loss) groups as described in the text. Line represents best fit. **(B)** Box and whisker plots show statistically significant differences between these groups. Open circle represents suspected outliers, and numbers indicate the subject identification of the suspected outlier. Further comparison between these groups showed that those performing better in nose were younger **(C)** and exhibited better (lower) pure tone thresholds **(D)**. There were no clinically significant differences word recognition in quiet between these groups **(E)**. Persons performing better in the presence of background noise also exhibited more robust DPOAE SNRs **(F)** and lower DPOAE thresholds **(G)**. This group also exhibited lower CAP amplitude at 40 dB SL (**H**; compare with the normal line in Figure 6A), longer CAP latencies **(I)**, and lower CAP thresholds **(J)**. ^*^Statistically significant difference between groups.

The distribution of QSIN scores were roughly divided in half at 0 SNR Loss (Figure 9A), where 25 subjects performed better in background noise (Normal SIN) and 28 subjects performed worse in background noise (Poorer SIN). J-T testing showed that the group performing better in background noise had statistically significantly lower QuickSIN scores (QSIN = −1.0 ± 0.19 SNR loss vs. 3.4 ± 0.43 SNR loss), which provides confidence that there is a statistically significant difference (*p* = 0.000) in performance in background noise between these groups (Figure 9B). Subjects performing better in background noise were statistically significantly younger (mean = 39.7 ± 2.71 years vs. 52.6 ± 3.71 years, *p* = 0.022; Figure 9C) and had statistically significantly lower audiometric thresholds (hfPTA = 10.0 ± 2.29 dB HL) compared to subjects with poorer performance in background noise (mean = 33.6 ± 2.71 dB HL PTA, *p* = 0.00), with the latter group exhibiting a mild sloping SNHL above 1 kHz (Figure 9D). There were no clinically significant differences in word recognition in quiet between these two groups when NU-6 word lists were presented at any sensation levels (Figure 9E).

J–T testing showed that subjects exhibiting better speech discrimination in the presence of background noise also exhibited statistically significantly greater DPOAE SNRs from 1 to 6 kHz (maximum difference of 10.77 dB SNR at 4 kHz, *p* = 0.00; Figure 9F) and lower DPOAE thresholds from 1 to 4 kHz (maximum difference of 15.11 dB SPL at 3 kHz, *p* = 0.00; Figure 9G) compared to subjects performing poorer in background noise. This data indicates that persons who performed better in background noise exhibited more robust DPOAE responses and suggests that loss of OHC function may diminish speech recognition in the presence of background noise.

Interestingly, the results suggest that the AN may also play a role in speech discrimination in the presence of background noise when OHC function is also abnormal. Similar to the Normal group in Figure 6, the Normal SIN group exhibited a statistically significant direct correlation between QSIN scores and CAP amplitude at presentation levels below 50 dB SL (τ_b_ = 0.285, *p* = 0.005 at 40 dB SL; Figure 8C), and a non-significant inverse correlation at higher presentation levels (maximum τ_b_ = −0.111, *p* = 0.319 at 60 dB SL; Figure 8D). J–T testing showed that while on average, those individuals performing better in background noise (i.e., lower QSIN scores) exhibited higher CAP amplitudes at louder presentation levels (above 50 dB SL), the variability in CAP amplitude also increased at higher presentation levels, particularly in the Normal group, so that no statistically significant differences in CAP amplitude existed between these groups (Figure 9H). In contrast, those individuals who performed better in background noise exhibited smaller CAP amplitudes when the tone pips were presented at lower presentation levels (below 50 dB SL), although this difference was only significant at 40 dB SL presentation levels (−0.178 μV difference, *p* = 0.01).

SIN performance exhibited a statistically significant inverse correlation with CAP latency (maximum τ_b_ = −0.423, *p* = 0.000 at 40 dB SL; Figure 8E) and CAP threshold (maximum τ_β_ = −0.215, *p* = 0.038 at 40 dB SL; Figure 8D). J–T testing demonstrated that those persons performing better in background noise exhibited statistically significantly longer CAP absolute latencies at presentation levels ranging from 40 to 70 dB SL (maximum difference of 0.553 ms at 40 dB SL, *p* = 0.07; Figure 9I) and statistically significantly higher CAP thresholds (mean = 34.8 dB SL) compared to those performing poorer in background noise (25.9 dB SL, *p* = 0.03; Figure 9J).

Therefore, the general pattern of AN function for persons with poor SIN performance (higher CAP amplitude at low presentation levels, shorter latencies, lower threshold) more closely resembled the AN dysfunction observed in the Minimal-Moderate hfPTA groups in Figure 6 where the OHCs were damaged rather than the AN response measured from cochleas with normal OHC function (higher CAP amplitudes, longer CAP latencies) shown in Figures 7A,B. Taken together, this data suggests that the those subjects with poor SIN performance may exhibit AN dysfunction as well as OHC dysfunction.

To test this theory, a linear mixed effects model (Bramhall et al., 2015) was generated to predict the relative contributions of OHC and AN activity on SIN performance. This model predicted that the main effects of DPOAE amplitude and DPOAE threshold had significant effects on QSIN scores (*p* = 0.01 and *p* = 0.04, respectively; Table 1), but the main effects of CAP amplitude did not (*p* = 0.25). Furthermore, this model also predicted that the interaction between DPOAE and CAP amplitudes did not have a significant effect on QSIN scores (*p* = 0.37), nor did the interaction between DPAOE thresholds and CAP amplitudes (*p* = 0.49). Furthermore, there were no statistically significant main effects or interaction effects on QSIN scores when factoring in CAP amplitudes at higher presentation levels (i.e., 60 dB SL), CAP latencies at any presentation level, or CAP thresholds into this model (data not shown). These results suggest that OHC function, rather than AN function, is a statistically significant predictor of SIN.

**Table 1:** **Linear mixed effects model of speech perception in noise**.

**Dependent variable**	**Predictor (Fixed effects)**	**Coefficient**	**Standard error**	***p-*****values**
QuickSIN (SNR loss)	Intercept	2.661 (dB SNR Loss)	1.081103	**0.03**
	DOPAE amplitude (4 kHz)	−0.222 (dB SNR Loss/dB SNR)	0.073916	**0.01**
	DOPAE threshold (3 kHz)	0.071 (dB SNR Loss/dB SPL)	0.032974	**0.04**
	CAP amplitude (40 dB SL)	1.846 (dB SNR Loss/μV)	1.533834	0.25
	DPOAE ^*^ CAP amplitudes	0.159 (dB SNR Loss/dB SNR ^*^ μV)	0.168321	0.37
	DPOAE threshold ^*^ CAP amplitude	−0.589 (dB SNR Loss/dB SPL ^*^ μV)	0.083386	0.49

*Bold text indicates a statistically significant p-value ≤ 0.05*.

### SIQ at or near threshold is correlated with OHC function

**Figure 10:**
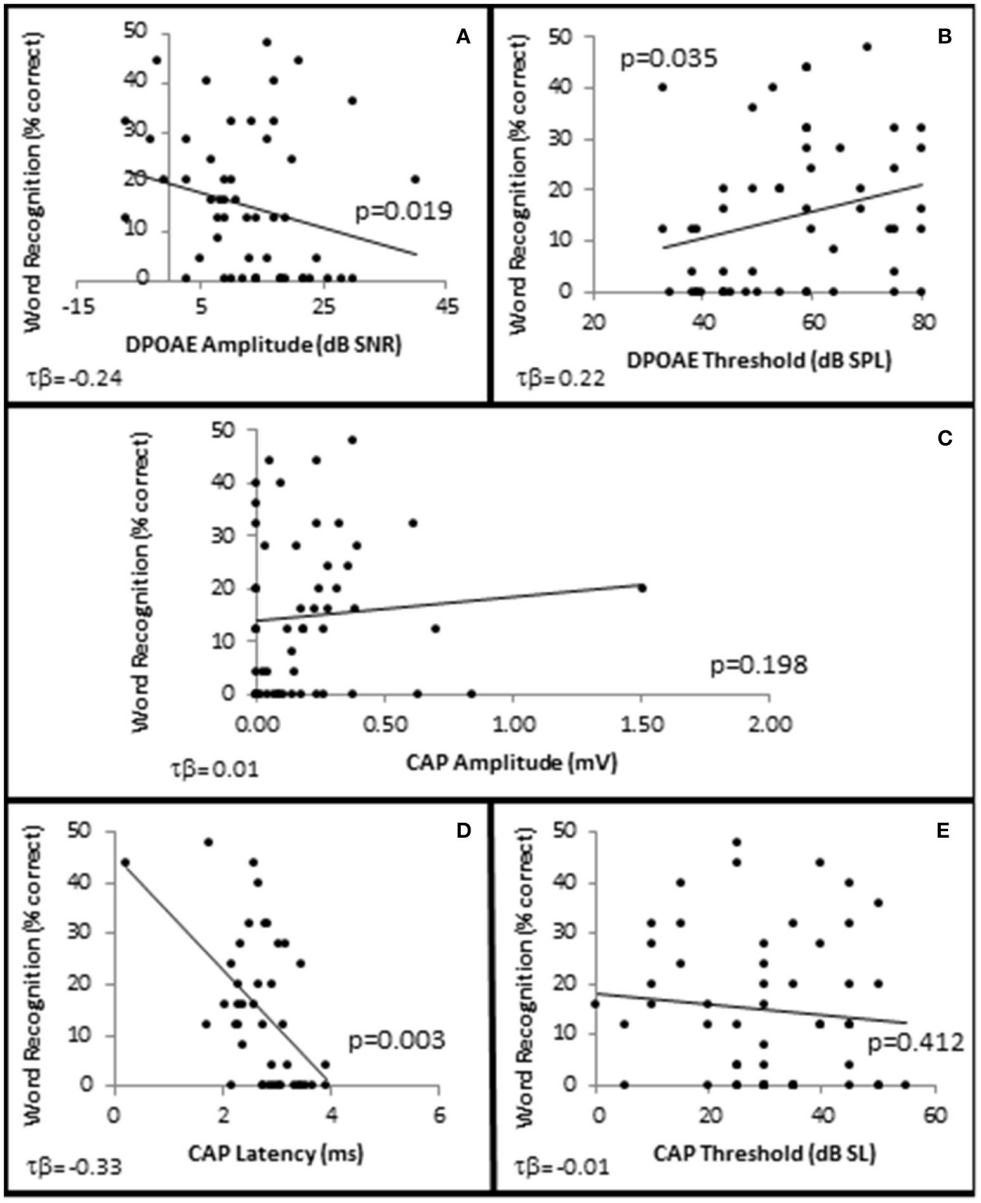
**SIQ at or near threshold is correlated with OHC function**. Distribution of WRSs in quiet from all subjects presented at 0 dB SL as a function of DPAOE amplitude **(A)** and DPAOE threshold **(B)** at 4 k Hz; as well as AN function measured by CAP amplitude **(C)**, and latency **(D)** in response to 4 kHz tone pips presented at 40 dB SL, and CAP thresholds **(E)**. Lines represent best fit (linear).

In order to examine whether OHC and or AN function played a role in speech recognition in quiet, subjects were presented NU-6 word lists at equivalent SLs and the subjects' WRSs were correlated with OHC and AN function. The results showed that presentation Levels between 10 and 40 dB SL failed to yield clinically significant differences in any metric (data not shown). However, WRS presented at or near threshold was correlated with OHC function (Figure 10).

**Figure 11:**
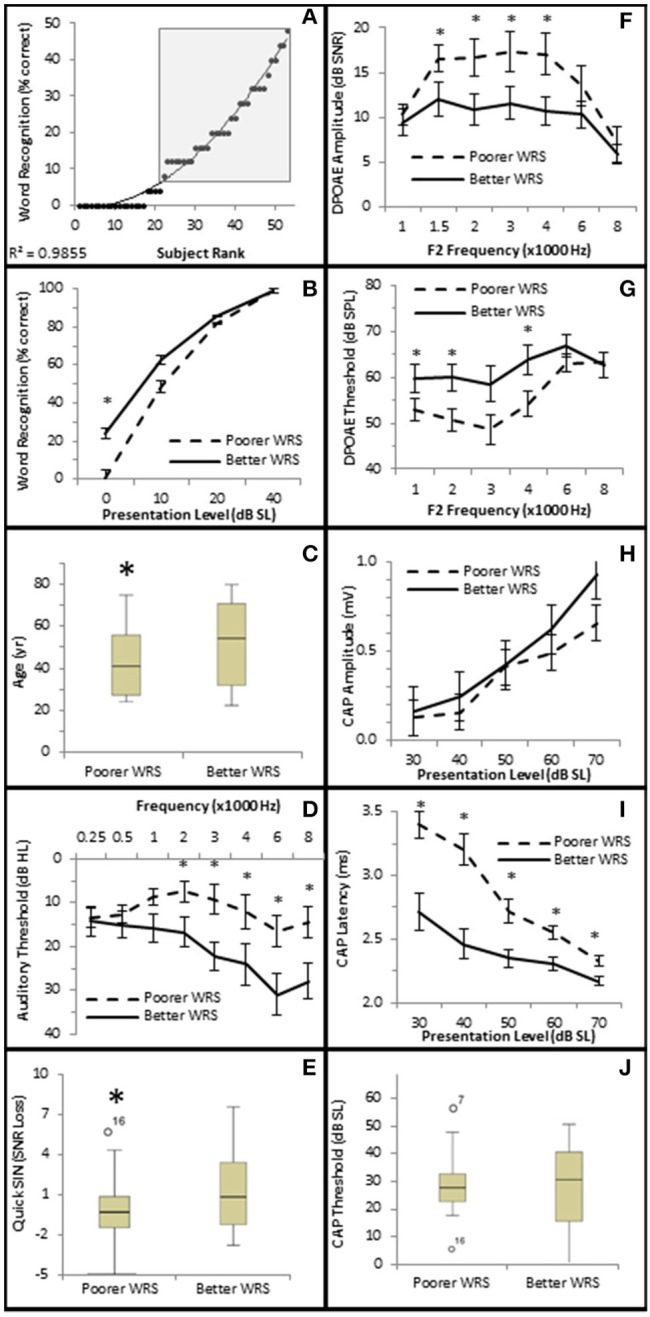
**Subjects with better SIQ performance at or near threshold exhibit OHC dysfunction. (A)** Subjects were ranked by Word Recognition scores presented at 0 dB SL, and were divided into either Poorer (WRS <8% correct) or Better (WRS > 4% correct, shaded box) groups as described in the text. Diagonal line represents best fit. Comparison between these groups showed that those performing better in quiet at or near threshold exhibited statistically significant improved WRS at low presentation levels **(B)**, were older **(C)**, with poorer (higher) pure tone thresholds **(D)**, and poorer (higher SNR Loss) speech in noise performance **(E)**. Persons performing better in quiet at or near threshold also exhibited diminished DPOAE SNRs **(F)** and higher DPOAE thresholds **(G)**, suggesting the OHC function plays a role in speech in quiet at or near threshold. This group failed to exhibit statistically significant differences in CAP amplitude **(H)** or threshold **(J)**. However, this same group exhibited statistically significantly lower CAP latencies **(J)**. Open circles in **(E,J)** represents suspected outliers, and numbers indicate the subject identification of the suspected outlier. ^*^Statistically significant difference between groups.

To further investigate this, subjects were divided into one of two groups depending upon their performance on the NU-6 word list presented at or near their individual thresholds (0 dB SL). Based on the 95% critical difference limits of the measured results listed in Thornton and Raffin (1978) (see in Table 5 of this reference), subjects were divided into either a poorer performing group who either scored 0% or 4% (one word) correct (Poorer WRS group, *n* = 21), or a better performing group who scored between 8 and 48% correct (Better WRS group, *n* = 32; Figure 11A). The 95% critical difference limits of Thornton and Raffin (1978) revealed a statistically significant performance gap between the Poorer WRS and Better WRS groups at presentation levels of 0 dB SL (0.8 ± 0.35% correct vs. 24.0 ± 1.2% correct). J–T testing of this same data similarly revealed a statistically significant difference between these groups (*p* = 0.000; Figure 11B). J–T testing between these groups at 10 dB SL also showed a difference between the Poorer WRS and Better WRS groups (43.8 ± 4.3 vs. 62.8 ± 2.9% correct), however, these results were not clinically significant when using the binomial modeling of speech discrimination typically used in the clinic (Thornton and Raffin, 1978).

Those subjects performing poorer in quiet near threshold were statistically significantly younger subjects (40.3 ± 3.38 years vs. 49.8 ± 3.27 years, *p* = 0.047; Figure 11C), with better high frequency hearing thresholds (maximum difference at 6 kHz of 14.5 dB HL, *p* = 0.027; Figure 11D). These two groups also exhibited statistically significant differences in hearing in the presence of background noise. Those subjects exhibiting poorer WRS at or near threshold performed better on the QSIN (0.2 ± 0.45 SNR loss) than those exhibiting better WRS in quiet at or near threshold (2.0 ± 0.53 SNR loss, *p* = 0.045; Figure 11E), suggesting that different mechanisms may be involved in speech perception in quiet at or near threshold and in the presence of background noise.

SIQ at or near threshold was inversely correlated with OHC function, where WRS was negatively correlated with DPAOE SNR (maximum τb = −0.237, *p* = 0.019 at 4 kHz; Figure 10A) and positively correlated with DPAOE threshold (maximum τb = 0.216, *p* = 0.035 at 2 kHz; Figure 10B). J–T testing showed statistically significant differences in OHC function between these groups with the Better WRS group exhibiting lower DPOAE amplitudes (maximum difference of −6.38 dB SNR at 4 kHz, *p* = 0.005; Figure 11F) and higher DPOAE thresholds (maximum difference of 9.43 dB SNR at 4 kHz, *p* = 0.009; Figure 11G) than their poorer performing counterparts.

While Better WRS performing groups on average exhibited larger CAP amplitudes (Figure 11H) and lower CAP thresholds (Figure 11J), neither of these effects were statistically significant. However, one component of the AN response correlated with word recognition at or near threshold in quiet. There was a statistically significant inverse correlation between WRS and CAP latency (maximum τ_b_ = −0.334, *p* = 0.003 at 40 dB SL; Figure 10D). J–T testing showed that the Better WRS group exhibited shorter wave I absolute latencies (maximum difference of 0.74 ms at 40 dB SL, *p* = 0.000; Figure 11I) than the group performing poorer on WRS at or near threshold. This data suggests that persons with diminished OHC activity may perform better in quiet at or near thresholds when stimuli are presented at equivalent SLs.

## Discussion

The overall goal of this study was to investigate OHC and AN function in regards to speech discrimination in quiet and in presence of background noise. Animal studies have speculated that the multiple AN fiber innervation of individual IHCs may function in complex listening situations such as speech detection in the presence of background noise (Schuknecht and Woellner, 1953; Kujawa and Liberman, 2009; Makary et al., 2011; Furman et al., 2013), however this theory is difficult to test using animal models. Furthermore, AN fiber density has been correlated to the wave I amplitude of the auditory brainstem response (ABR) in animal studies where OHC integrity has been preserved (Kujawa and Liberman, 2009; Lin et al., 2011), suggesting that wave I amplitude may be used as a tool to measure AN density. This paper attempted to determine whether ECochG CAP amplitude, which is synonymous with wave I of the ABR, correlated with SIN or SIQ in human subjects, and also to determine whether OHC function measured by DPOAEs contributed to these complex listening tasks.

A previous study using linear mixed effects models in humans similarly found that SIN was correlated to an inverse interaction between ECochG CAP amplitude and SNHL, while ECochG CAP amplitude had no effect on SIQ (Bramhall et al., 2015). This current study supports the later observation (Figures 10C, 11H). The aforementioned study used a 4 kHz tone pip presented at 70 dB SPL to evoke the CAP and found that subjects exhibiting both better audiometric thresholds and high ECochG CAP amplitudes performed better on the QSIN. That study found inverse correlations between age and CAP amplitude, SNHL and CAP amplitude, SIN performance, and SNHL, however that study found no direct correlation between ECochG CAP amplitude and SIN performance. Rather, SIN performance was dependent on the inverse interaction between SNHL and ECochG CAP amplitude, where persons who exhibited both lower CAP amplitudes and poorer audiometric thresholds were found to have performed poorly in the presence of background noise. One possible reason why Bramhall et al. (2015) failed to find statistically significant differences between ECochG CAP amplitudes and SIN performance was the high variability in CAP amplitude, particularly among persons with PTAs <12.5 dB HL. Another possible factor could have been that both SIN performance and ECochG CAP amplitude were so strongly correlated with SNHL that the degree of hearing loss could mask differences in these variables. A third possibility could be that CAP amplitudes are not correlated with SIN in humans.

This current paper used graded SL presentations in order to correct for the effect of the degree of SNHL on ECochG CAP amplitude. These results suggest that a loss of tuning in pathological ears leads to level dependent changes in ECochG CAP amplitudes, shorter CAP latencies, and lower (better) CAP thresholds when stimuli were presented at equivalent SLs. Furthermore, these results indicate that normal OHC function is required for optimal SIN performance.

### Effects of diminished cochlear tuning on ECochG CAP amplitudes

**Figure 12:**
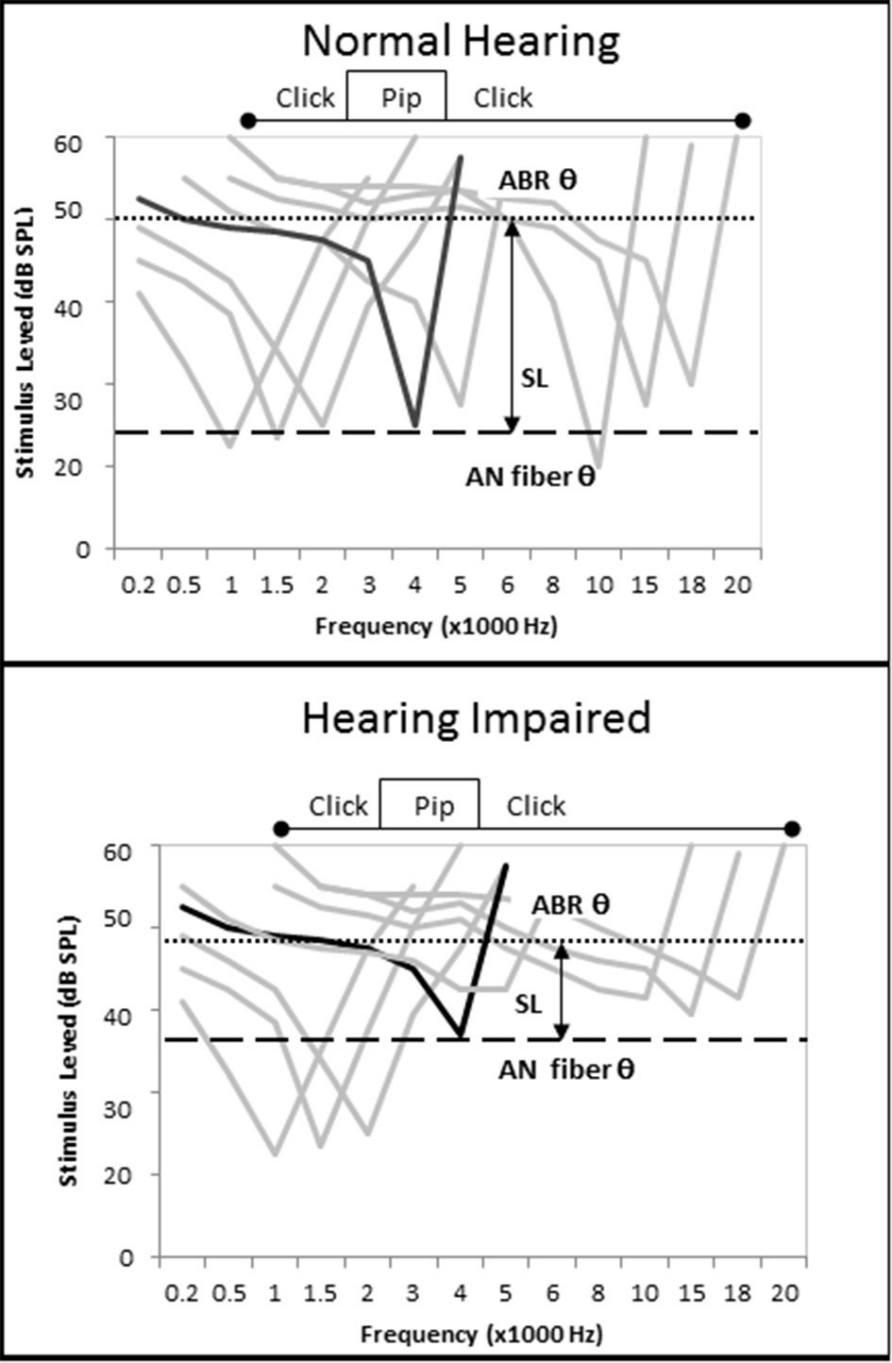
**Loss of cochlear tuning results in SL-dependent changes in CAP amplitude, shorter CAP latencies, and lower CAP thresholds. (Top)** Hypothetical AN fiber tuning curves in a listener without otopathology illustrating the CF of a fiber tuned to 4 kHz (black) and off-tuned fibers (gray). The difference between the AN fiber threshold and ECochG threshold, which can be imagined as a subject's SL, occurs because the level must be raised by 25 dB SPL to recruit enough off-tuned AN fibers to evoke a CAP. When the tone pip is presented at a low level, relatively few AN fibers are recruited, so CAP amplitude is low. When the tone pip is presented at a higher level, the stimulus acquires the acoustic characteristics of a click and more off-fibers are recruited and CAP amplitude increases (compare with Figure 6A). **(Bottom)** OHC damage leads to increased audiometric thresholds, micromechanical distortions to BM vibrations, and changes in AN fiber tuning that include an elevation of AN fiber threshold and a broadening of off-fiber tuning. When a 4 kHz tone pip stimulus is presented at a low SL, the net effect of this otopathology is a hypersensitivity of off-fiber tuning that leads to recruitment of more off-tuned ANF fibers, lower CAP thresholds, shorter CAP latencies, and higher CAP amplitudes. At higher presentation levels where the stimuli acquires characteristics of a click, the otopathology leads to a decrease in the number off-tuned AN fibers that can be recruited so the CAP amplitude does not increase as drastically as seen in the non-pathological ear (Figure 6A), and a CAP amplitude decreases in comparison to the non-pathological ear.

There were contrasting results related to ECochG CAP amplitude in this study depending on the intensity level of the tone pip used to evoke the CAP and the degree of SNHL exhibited by the subjects. On average, increased SNHL resulted in diminished ECochG CAP amplitudes at higher stimulus levels, however the opposite effect was observed at presentation levels below 50 dB SL (Figure 6A). There could be different causes for this observation. Considering first the Normal hfPTA group (solid line in Figure 6A), increasing presentation level likely increased the frequency spectrum of the stimuli, which may in turn affect the CAP amplitude (Figure 12). At lower presentation levels, the stimuli consisted of tone pips with limited frequency spectrum, but as the presentation intensity increased above 40 dB SL (Normal group) the frequency spectrum of the stimulus and the population of AN fibers activated by that stimulus would be expected to increase to more closely resemble a click (Pfeiffer and Kim, 1975). Therefore, higher presentation levels would recruit more AN fibers and increase the CAP amplitude and variability, which is seen in the normal group in Figure 6A. Placing the variability in CAP amplitude aside for the time being, it could be assumed that an increase in AN fiber activation would lead to increased CAP amplitudes at louder presentation levels, which is the general trend in this figure. This may explain the observation in Figure 9H, where persons with normal SIN performances who also have normal hfPTAs (Figure 3C) and normal OHC function (Figure 5) exhibit higher CAP amplitudes at louder presentation levels than persons with SNHL.

On the other hand, lower presentation levels suggest the opposite effect. In these cases, persons in the Normal hfPTA group exhibited lower CAP amplitudes than those with moderate SNHL (Figure 6A), and also performed better in noise (Figure 9H), the latter of which is expected clinically in a person with normal hearing. This data shows that as hearing impairment increased from minimal to moderate SNHL, the amplitude of CAP increased and the SIN performance decreased in response to lower SL presentations. This increase in CAP amplitude at low SL exhibited by persons with SNHL may be attributed to a combination of two factors. First, a loss of tuning caused by OHC dysfunction would be expected to lead to a broadening of the BM vibration at a given frequency (Liberman and Dodds, 1984). This loss of tuning, or loss of the cochlear amplifier, should result in a change of AN fiber tuning, where the characteristic frequency (CF) of a given AN fiber becomes elevated (threshold elevation) and its tuning curve becomes broader and exhibits a hypersensitivity in adjacent AN fibers with higher CFs (Liberman and Dodds, 1984). Since auditory evoked responses are more sensitive (lower thresholds) in the high frequency regions of the cochlea (Goldstein et al., 1971), this shift in tuning to a higher frequency may recruit more AN fibers to fire for a given stimulus (Pfeiffer and Kim, 1975), and would increase CAP amplitude (Figure 6A), decrease CAP latency (Figure 6B), and lower CAP threshold (Figure 6C). One way to explain this data is with linear systems theory (Goldstein et al., 1971; Liberman and Dodds, 1984; Ruggero, 1994; Henry et al., 2011), where the OHCs function as a bank of frequency filters that fine-tune the response of not only the BM, but the AN fibers as well. This fine-tuning means that fewer AN fibers are recruited in a non-pathological ear for a low SL presentation, and the CAP amplitude is relatively lower, CAP latency is relatively longer, and CAP threshold is relatively higher. Loss of the OHC filter function results in a broadening of the region of AN activation even for low SL presentations that is reflected in the increase in CAP amplitude in those persons with OHC damage (i.e., Minimal to Moderate SNHL groups in Figure 6A).

The second process causing the increase in CAP amplitude at low SL exhibited by persons with SNHL may be due to the SPL of the stimuli needed to elicit an AN response. For a normal cochlea, a 4 kHz tone pip presented at 40 dB SL (i.e., 40 dB HL in a normal ear) would elicit activation of a select population of AN fibers whose CFs are close to this place of resonance on the BM. The loss of OHCs would lead to a broadening of BM resonance, a change in AN tuning, and a decrease in PTA. In these patients, a tone pip presented at 40 dB HL may be sub-threshold and not activate enough AN fibers to elicit a CAP, and so the intensity level would have to be raised to reach CAP threshold. In such cases, those persons with a moderate SNHL would need a 40 dB SL stimulus that is presented at a higher SPL (i.e., 70 dB HL) to recruit enough AN fibers to elicit a CAP. This loud presentation level would lead to a broader area of BM resonance and would activate a larger population of AN fibers (Pfeiffer and Kim, 1975) that would result in higher CAP amplitudes. In this case, the fact that they are low presentation levels on the SL scale may obfuscate the fact that a more intense signal is required to elicit a response. This observation is born out in Figure 6A where persons exhibiting SNHL have a comparatively more linear growth function than the non-linear function of the Normal hfPTA group.

The CAP amplitude-intensity function, therefore, can be used to estimate two different sites of lesions of the 8th cranial nerve. At lower presentation levels, the 4 kHz tone pip may be measuring specific damage to cells that affect the tuning to this frequency, while at higher presentation levels, this stimulus loses its frequency specificity but measures the activity of a greater population of the AN fibers. Therefore, measuring AN activity using the SL based CAP amplitude-intensity function is a way to measure both site specific and more global AN dysfunction. That being said, measuring CAP amplitude in humans has some inherent complications.

Unlike laboratory animals, ECochG CAP, and ABR amplitudes are notoriously variable in humans (Gorga et al., 1985; Winzenburg et al., 1993; Burkhard et al., 2007; Hall, 2007). Causes of this variability include placement of the recording electrode and physiological noise inherent in these recordings. The typical ECochG recording methods include a trans-tympanically needle electrode placed on the base of the cochleae, a wick electrode placed on or near the tympanic membrane as in this current study, or gold foil wrapped triptodes placed near the opening of the external auditory meatus. Bramhall et al. (2015) used triptodes and also found a large variability in ECochG CAP amplitude, and the decision to move to a wick electrode near the tympanic membrane for this study was an attempt to address this variability. While the magnitude of the amplitudes had increased as the recording was conducted closer to the cochlea in the paper presented here, there were no statistically significant changes in the variability of the amplitude between triptodes and wick electrode methods (data not shown). This observation is born out in the literature as well (Winzenburg et al., 1993). Another source of variability has been attributed to the presence intrasubject noise consisting of both electromyographical and electroencephalographical artifacts (Zvonar et al., 1974), which is diminished in animal studies because animals are normally sedated during ABR testing while humans are typically not sedated. Further studies on sedated humans could be conducted to determine whether sedation leads to a lower variability in CAP amplitude as observed in animal studies. Sex genotype also plays a role in ABR amplitude and latency (Don et al., 1993). However, differences in sex should not affect this analysis because the subjects were divided in groups based on their behavioral or physiological responses irrespective of their sex. Regardless, the intake questionnaire of this study didn't differentiate between gender identity and sex genotype, the latter of which presumably exerts a stronger effect on CAP amplitudes than the former. Therefore, a complete analysis of sex genotype/gender effects on these findings would be warranted in future studies.

Finally, a recent study has proposed using the ratio between the summating potential (SP) and action potential (AP) of ECochG CAP to estimate AN dysfunction in humans (Liberman et al., 2016). Reanalysis of the data presented in this paper utilizing the AP/SP analysis described in Liberman et al. (2016) showed that SP/AP ratio had no significant correlations to either SIN or SIQ described in this paper (data not shown). The difference between this current paper and the aforementioned paper could be due to the different methods of SIN testing. Here, the QSIN, which consisted of target sentences presented in increasing levels of background speech babble, was used because it has face validity for clinical applications. However, Liberman et al. (2016) saw significant differences in SIN performance between musicians and non-musicians when using a more complex SIN protocol consisting of 45% or 65% time compressed NU 6 word lists presented with 0.3 s reverberation and in ipsilateral narrowband noise. This is a more complex task that may be required to detect changes in AN dysfunction and which may or may not have translational correlations related to noise exposure or AN integrity. A comparative analysis between these different SIN assessments is warranted in future experiments.

### Effects of diminished cochlear tuning on CAP latency and threshold

In contrast to amplitude, absolute or inter-peak latency is a less variable metric and is used clinically for ABR analysis in humans (Hecox and Galambos, 1974). Factors that contribute to ECochG CAP latency include the cochlear transport time (Don et al., 1993), which is influenced by passive properties of the basilar membrane such as the stiffness gradient and mass loading; cochlear filter build-up time, which involves the “cochlear amplifier” processes (Davis, 1983), where OHC depolarization sharpens the tuning of the basilar membrane and shifts the frequency of resonance more apically; the neurotransmission time that involves the summation potential, AN synchrony, and frequency characteristics of the AN fibers; and frequency and intensity characteristics of the acoustic stimuli which would influence all of these processes (reviewed in Don et al., 1998). It has been proposed that OHC dysfunction results in the loss of the cochlear filter build-up time, which would result in the decrease in ABR latency observed in patients with cochlear (sensory), as opposed to retrocochlear, hearing loss (Don et al., 1998; Lichtenhan and Chertoff, 2008; Henry et al., 2011). As mentioned previously, OHC damage is also known to cause hyper-sensitivity in the tail regions of AN fiber tuning curves (Liberman and Dodds, 1984), which is theorized to lead to a shift in AN fiber tuning to higher frequencies that would decrease the latency in hearing impaired individuals (Goldstein et al., 1971; Lichtenhan and Chertoff, 2008; Strelcyk et al., 2009; Henry et al., 2011). As mentioned above, increasing the stimulus intensity may also increase the population of active AN fibers, which would be expected to decrease the latency as well. Therefore, CAP latency may be affected by OHC dysfunction, altered AN tuning, and stimulus variables.

The data presented in Figure 6B indicates that ECochG CAP latency decreases with increasing SNHL when the stimulus is presented at equivalent SLs. This data is the opposite of many studies that found either increased age or increased SNHL resulted in an increase in ABR wave latency in response to click evoked stimuli (Attias and Pratt, 1984; Gorga et al., 1985; Gourevitch et al., 2009). However, those studies used broad band stimuli presented at a constant presentation level and did not control for the effect of hearing loss on the stimulus level. In order to correct for an individual's hearing threshold on stimulus intensity, this current study presented stimuli relative to their individual audiometric thresholds, or SL. The results presented in this paper indicate that CAP latency is inversely proportional to an individual's hearing loss when the stimulus is presented at equivalent SLs. This observation is similar to previous studies where sensory hearing loss was correlated with a decrease in absolute CAP (Lichtenhan and Chertoff, 2008) and ABR wave V latency in humans (Don et al., 1998; Strelcyk et al., 2009; Scheidt et al., 2010) and a decrease in absolute wave I amplitudes and latencies in chinchillas (Henry et al., 2011) when narrow band stimuli (derived-band ABR and tone burst, respectively) were presented at equivalent SLs.

Our data indicates a general trend of an inverse relationship between ECochG CAP amplitude and latency at both low and high presentation levels (Figures 6A,B) when OHC function is normal. These data also suggests that ECochG CAP latency is a more reliable metric than CAP amplitude to differentiate between normal and abnormal SIN (Figure 9I) and SIQ (Figure 11I) performance. ECochG CAP amplitude is more variable compared to CAP latency (Figure 1), and different presentation levels result in changes in relative amplitude (Figures 6A, 9H, 11H). In comparison, ECochG CAP latency is a more reliable predictor of SNHL, SIN, and SIQ. However, while wave I amplitude has been correlated to AN density in animal models, the contribution of AN fiber density on wave I latency has not been determined and further animals studies examining this are warranted.

Similar to latency, ABR thresholds, rather than amplitudes, are routinely used clinically in humans but not without criticism (Eggermont, 1982). Here, we demonstrated that ECochG CAP thresholds decreased with increasing hearing loss at equivalent presentation levels (Figure 6C). As explained in the preceding discussion, stimulus effects, and decreased tuning would explain the decrease in ECochG CAP thresholds when constant stimulus intensity is used. In non-pathological ears, there is typically a 25 dB SPL difference between AN fiber threshold and CAP threshold (Ngan and May, 2001; Henry et al., 2011). Animal data has shown that hearing impairment causes an upward compression of both AN fiber thresholds (Liberman, 1978) and ABR threshold range (Ngan and May, 2001) that may reduce the difference between AN fiber and CAP thresholds and act to lower ABR thresholds in hearing impaired subjects. Support for this theory is presented in this paper when considering that hearing impaired subjects (older subjects with poorer audiometric thresholds and poorer OHC function) exhibit lower CAP thresholds that their normal hearing counterparts when stimuli are presented on an SL scale (Figure 9J).

### Anatomical correlates of SIN and SIQ

The effect of SIN performance on ECochG CAP amplitude was not as initially expected. The initial hypothesis was that CAP amplitude, which correlates with AN fiber density when using click stimuli, would directly correlate with SIN performance. However, when controlled for normal OHC function, those individuals with higher CAP amplitudes (Figure 7A) failed to exhibit statistically significant differences in either SIQ (Figure 7E) or SIN (Figure 7F) performance compared to those exhibiting lower CAP amplitudes. This suggests that SIN performance is not correlated with CAP amplitude, and therefore SIN is not correlated to AN fiber density. Alternatively, it could be that the differences in amplitude between these two groups is too similar and needs to be greater than we defined in this paper to show a statistically significant difference in SIN performance. Further studies in a larger population of persons with normal DPOAEs and diminished CAP amplitudes may be required to definitively determine whether reduced CAP amplitudes correlated with reduced SIN performance. As mentioned previously, it could be that more challenging SIN assessments, such as time compressed speech in reverberation, may find a statistically significant difference between these groups. However, these current results suggest that when controlled for OHC damage, CAP amplitude by itself is not a predictor of either SIN or SIQ performance.

Since ECochG CAP latency includes components of OHC function and neural transmission, comparing CAP latency and DPOAE results may help to determine whether OHCs or the AN contribute to the behavioral response. For instance, the data presented in Figure 6B indicates there is not a significant difference in latency between the Normal and Minimal SNHL groups, whereas Figure 5 indicates that OHC amplitudes and OHC thresholds are diminished in the Minimal SNHL group. This suggests that persons exhibiting minimal SNHL exhibit OHC dysfunction rather than statistically significant AN dysfunction. The data also suggests that speech in noise performance is correlated with both OHC function (Figures 8A,B, 9F,G) and CAP latency (Figures 8E, 9I), with persons performing better in the presence of background noise (lower QSIN score) exhibiting more robust OHC responses (higher DPOAE SNRs and lower DPOAE thresholds), and longer CAP latencies (Normal group) than those performing poorer in background noise. These results suggest that the AN may play a role in SIN, however as mentioned above, several variables contribute to CAP latency and so it cannot be concluded that AN dysfunction contributes to SIN by analyzing CAP latency alone.

These results provide scant evidence that AN integrity is a major variable contributing to SIN performance. The observation that persons performing better in noise also exhibit both lower CAP amplitudes at lower SLs (Figures 8C, 9H) and lower CAP thresholds (Figures 8F, 9J), which are profiles consistent with normal hearing, bolster the hypothesis that the AN also plays a role in SIN, however the CAP amplitude data was only significant at one low presentation level (40 dB SL; Figure 9H). Further evidence of both OHC and AN involvement in SIN performance can be seen when analyzing loss of function in SNHL patients. These results suggest that OHC dysfunction may occur in minimal degrees of SNHL (Figure 5), while AN dysfunction may not be statistically significant until greater degrees of SNHL (mid SNHL for CAP latency effects, and moderate SNHL for CAP amplitude and threshold effects; Figure 6). Furthermore, there are no statistically significant differences in SIN between Normal hfPTA and Minimal SNHL groups (Figure 3C), but statistically significant differences in SIN performance first appear in the Mild SNHL group. These results suggest the possibility that AN dysfunction may play a role in decreased SIN performance. However, the linear mixed effects model, which is a statistical approach that incorporated the variances associated with every variable measured in this study into a single statistical model, showed that CAP amplitude failed to have a statistically significant correlation with SIN performance (Table 1). Furthermore, this model showed that both DPOAE amplitude and DPAOE thresholds are correlated with QSIN scores, which suggests that OHC function is a primary variable contributing to SIN performance using the QuickSIN.

Similarly, results from the SIQ study can be used to differentiate between the OHC and AN components in CAP latency measures. As shown in Figure 10, persons with better WRS in quiet at or near threshold exhibited poorer OHC function (Figures 10A,B, 11F,G) and shorter CAP latencies (Figures 10D, 11I), but exhibit equivocal CAP amplitudes (Figures 10C, 11H) and thresholds (Figures 10H, 11J). Since ECochG CAP latency encompasses both OHC and AN functions, and there are no differences in CAP threshold or amplitude between these groups, one conclusion could be that OHC dysfunction rather than AN (dys)function enhances SIQ performance at or near threshold. This can be explained by the normally sharp tuning of the BM and subsequent sharp tuning of the AN fibers through normal OHC function, which act as a bank of filters with an end result that limits SIQ performance at or near threshold. OHCs may act more like a filter bank at low presentation levels that enhances frequency sensitivity measured by pure tone thresholds (Figure 11D) but diminishes speech discrimination performance in quiet at or near threshold (Figure 11E). Another explanation for improved SIQ performance may be due to the increased presentation levels to those persons exhibiting SNHL. As can be seen in Figure 11D, on average those persons performing better in quiet also exhibit a sloping SNHL. Therefore, it could be that these persons utilize low frequency information for speech recognition in quiet at or near threshold. It is possible that both processes, OHC damaged filter function coupled with the increased stimulus levels, leads to enhanced SIQ performance at or near threshold. Further studies analyzing the correlation between slope and degree of SNHL would be helpful in describing the contributions of OHC dysfunction and signal level in SIQ performance.

There may be a behavioral correlate for this in humans. Some persons with SNHL also exhibit an unusual growth in the perception of loudness, termed loudness recruitment (Dix et al., 1948). The data presented here suggests that loudness recruitment may be acting at the level of the inner ear. Previous studies and have shown that OHC damage causes hypersensitivity in the tail region of the damaged AN fiber tuning curve, which has been interpreted to mean that one role of the OHC is to decrease the sensitivity of AN fibers tuned to adjacent CFs (Liberman and Dodds, 1984). In this sense, individual OHCs may function as a band-pass filter to dampen the stimulation of adjacent AN fibers. Loss of this function would lead to recruitment of adjacent AN fibers, which is consistent with the hypothesis that loudness recruitment is caused by OHC dysfunction (Moore, 2002). Furthermore, it may be that AN dysfunction also plays a role in this phenomenon. Since low spontaneous rate fibers are more susceptible to damage, they may be missing in this population and high spontaneous rate fibers, which function in quite backgrounds, are likely left intact (Furman et al., 2013). Therefore, it could be that the neural pathway in persons with SNHL is optimized for speech understanding in quiet at or near threshold.

### Unhidden hearing loss: profile of SNHL

It is well-documented that the standard audiogram in insufficient to adequately describe the underlying otopathology that causes SNHL (Merchant and Nadol, 2010). Proper definition of the functional roles of inner hair cells (IHCs), OHCs, and AN fibers is essential for the understanding of the cellular basis of audition. As important, biotechnologies using drug, cell based, or gene therapies aimed at regenerating hair cells or AN fibers (reviewed in Parker, 2011) will depend upon proper assessment of these cell types in order to identify the underlying otopathologies involved in SNHL. Improvements on hearing aid and cochlear implant technologies can also be made if the functions of the OHCs and AN fibers are known and are incorporated into their signal processing algorithms.

The data presented in Figure 5A suggests that OHC function is correlated with pure tone audiometry, and that even subjects with minimal high frequency SNHL may exhibit significant OHC damage. However, since a PTA between 15 and 25 dB HL is often considered within the normal range in adult humans, this finding illustrates an example of an undetected otopathology commonly known as “Hidden Hearing Loss (HHL),” which can be defined as an otopathology that is not recorded by the standard audiogram. Several subtypes of HHL have been described including auditory synaptopathy (Furman et al., 2013), auditory neuropathy (Starr et al., 1996; Makary et al., 2011), and OHC dysfunction (Gorga et al., 1997). This latter study examined DPOAEs in 806 subjects and found that OHC dysfunction was evident with a PTA of 20 dB HL or greater, which is within the 10–25 dB HL range typically used as clinically normal in human hearing. The data presented here argues for a lowering of the normative range cut-off from 20 dB HL (Gorga et al., 1997) to 15 dB HL and suggests that a minimal SNHL is a clinical presentation of an underlying OHC otopathology. As previously mentioned, recent studies have suggested that synaptopathy/auditory neuropathy can also occur in persons with PTAs below 25 dB HL (Liberman et al., 2016; Bramhall et al., 2017), even if the degree of impairment in this group is debatable (Prendergast et al., 2017). Therefore, there is growing evidence that the standard audiogram is a poor representation of the underlying otopathologies that cause SNHL and a holistic assessment may be more appropriate to better target future treatments.

From the data presented in this paper, the profile of SNHL can be defined as follows; a typically older person with a higher hfPTA, poorer OHC function (lower DPOAE SNR, higher DPOAE thresholds), poorer AN function (higher CAP amplitude at low presentation levels, lower CAP amplitude at higher presentation levels, shorter CAP latencies, lower CAP thresholds when controlled for SNHL), poorer SIN performance, and better SIQ performance at or near threshold. All of these characteristics can be easily measured using standard audiometric techniques presented in this paper. The data presented here indicates that those persons exhibiting SNHL perform better in quiet at or near threshold and may shed light on the anatomical correlates associated with increased sensitivity to loud sounds experienced by those afflicted with SNHL.

Rather than use a standard SPL scale, this paper used a SL scale in order to correct for the degree of SNHL. This scale is useful when considering the perception of the individual with hearing loss and may be useful in assessing therapies from the patient's perspective. For instance, while a 40 dB HL stimulus presented to a person with normal hearing is perceived, this same stimulus presented to a person with SNHL may be imperceived because it may be presented at a sub-threshold level. Therefore, the stimulus level must be increased in order for the hearing impaired listener to detect this signal; however, the signal being detected may be distorted, the loss of OHCs would lead to a broader region of the BM being deflected, and a larger population of AN fibers with modified tuning may be recruited to elicit a CAP. This may lead to a different listening experience between those with normal and pathological ears, which can be particularly problematic in terms in terms of amplification provided by hearing aids. The observation that persons with OHC dysfunction may actually perform better in quiet at or near threshold may be exploited in future technologies where speech in noise detection, rather than amplification, would be the targeted therapy.

## Ethics statement

This study was carried out in accordance with the recommendations of Steward St. Elizabeth's Medical Center Internal Review Board for Medical Research with written informed consent from all subjects. All subjects gave written informed consent in accordance with the Declaration of Helsinki. The protocol was approved by the Steward St. Elizabeth's Medical Center Internal Review Board for Medical Research.

## Author contributions

RH assisted in experimental design and performed Electroacoustic measurements. GE and SP assisted in experimental design, recruited subjects, and collected audiometric and DPAOE data. MP designed all experiments, wrote the IRB application, analyzed the data, and wrote the manuscript.

## Funding

This work was supported by departmental funds from the Department of Otolaryngology, Head and Neck Surgery at Steward St. Elizabeth's Medical Center in Boston, MA.

### Conflict of interest statement

The authors declare that the research was conducted in the absence of any commercial or financial relationships that could be construed as a potential conflict of interest.
